# Loss of FXR or Bile Acid-dependent Inhibition Accelerate Carcinogenesis of Gastroesophageal Adenocarcinoma

**DOI:** 10.1016/j.jcmgh.2025.101505

**Published:** 2025-03-24

**Authors:** Theresa Baumeister, Andrea Proaño-Vasco, Amira Metwaly, Karin Kleigrewe, Alexander Kuznetsov, Linus R. Schömig, Martin Borgmann, Mohammed Khiat, Akanksha Anand, Julia Strangmann, Katrin Böttcher, Dirk Haller, Andreas Dunkel, Veronika Somoza, Sinah Reiter, Chen Meng, Robert Thimme, Roland M. Schmid, Deepa T. Patil, Elke Burgermeister, Yiming Huang, Yiwei Sun, Harris H. Wang, Timothy C. Wang, Julian A. Abrams, Michael Quante

**Affiliations:** 1 Klinik für Innere Medizin II, Universitätsklinikum Freiburg, Freiburg, Germany; 2 Klinik und Poliklinik für Innere Medizin II, Technical University of Munich, Munich, Germany; 3 Spemann Graduate School of Biology and Medicine (SGBM), University of Freiburg, Freiburg, Germany; 4 Faculty of Biology, University of Freiburg, Freiburg, Germany; 5 Department of Nutrition and Immunology, Technical University of Munich, Munich, Germany; 6 Leibniz-Institute for Food Systems Biology, Technical University of Munich, Munich, Germany; 7 Bavarian Center for Biomolecular Mass Spectrometry, TUM School of Life Sciences, Technical University of Munich, Munich, Germany; 8 Department of Food Chemistry and Molecular Sensory Science, TUM School of Life Sciences, Technical University of Munich, Munich, Germany; 9 Department of Pathology, School of Medicine, Digestive Health Research Institute, Case Western Reserve University; Cleveland, Ohio; 10 Department of Internal Medicine II, Medical Faculty Mannheim, Heidelberg University, Heidelberg, Germany; 11 Systems and Synthetic Biology, Columbia University Medical Center, New York, New York; 12 Department of Medicine, Columbia University Irving Medical Center, New York, New York; 13 Herbert Irving Comprehensive Cancer Center, Columbia University, New York, New York

**Keywords:** Gastroesophageal Adenocarcinoma, Barrett Esophagus, Bile Acids, Farnesoid X Receptor, Obeticholic Acid

## Abstract

**Background & Aims:**

The incidence of Barrett esophagus (BE) and gastroesophageal adenocarcinoma (GEAC) correlates with obesity and a diet rich in fat. Bile acids (BAs) support fat digestion and undergo microbial metabolism in the gut. The farnesoid X receptor (FXR) is an important modulator of the BA homeostasis. When activated, FXR can inhibit cancer-related processes, and thus, it is an appealing therapeutic target. Here, we assess the effect of diet on the microbiota-BA axis and evaluate the role of FXR in disease progression.

**Methods:**

L2-IL1B mice (mouse model of BE and GEAC) under different diets, and L2-IL1B-FXR KO-mice were characterized. L2-IL1B-derived organoids were exposed to different BAs and to the FXR agonist obeticholic acid (OCA). The BA profile in serum and stool of healthy controls and patients with BE and GEAC was assessed.

**Results:**

Here we show that a high-fat diet accelerated tumorigenesis in L2-IL1B mice while increasing BA levels and altering the composition of the gut microbiota. Although upregulated in BE, expression of FXR was downregulated in GEAC in mice and humans. In L2-IL1B mice, FXR knockout enhanced the dysplastic phenotype and increased Lgr5 progenitor cell numbers. Treatment of murine BE organoids and L2-IL1B mice with OCA notably ameliorated the phenotype.

**Conclusion:**

GEAC carcinogenesis appears to be partially driven via loss or inhibition of FXR on progenitor cells at the gastroesophageal junction. Considering that the resulting aggravation in the phenotype could be reversed with OCA treatment, we suggest that FXR agonists have great potential as a preventive strategy against GEAC progression.

## Graphical abstract

**Figure:**
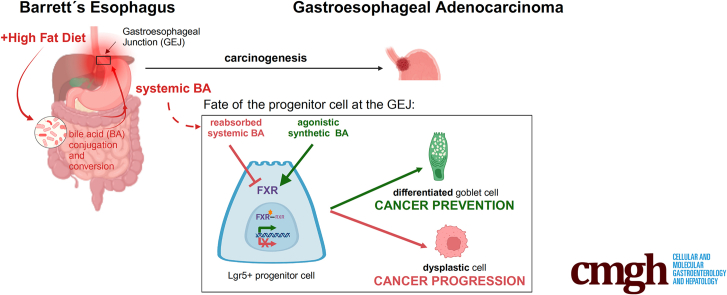

**Summary**

Although the inhibition of farnesoid X receptor is linked to disease progression, its activation is associated with cancer prevention. Therefore, exploring the potential of farnesoid X receptor as a therapeutic target has great clinical relevance in the context of gastroesophageal adenocarcinoma.

It has been proposed that chronic reflux of gastric and bile acids (BAs) triggers inflammation at the gastroesophageal junction (GEJ), leading to Barrett esophagus (BE) and gastroesophagel adenocarcinoma (GEAC).^1^ High-fat, Western-style diets and obesity have emerged as risk factors for GEAC^2^^,^^3^ and represent modifiable targets for cancer prevention. Western-style diet has been linked to the occurrence of precancerous gastrointestinal lesions,^4^ as it can provoke chronic inflammation in the gastrointestinal tract^5^ and directly affect the composition of gastrointestinal microbiota.

Thus far, studies relating diet and obesity to GEAC generally fail proving direct causality. In our transgenic mouse model of BE and GEAC (L2-IL1B), both, high-fat (HFD) and high-fructose diet led to a shift in the gut microbiota and accelerated GEAC carcinogenesis,^6^^,^^7^ suggesting that the intestinal microbiota has an impact on tissues distant to the gut. Gut bacteria are responsible for BA deconjugation and also for the conversion of primary to secondary bile acids,^8^ which can passively diffuse into the blood circulation across the colonic epithelial surface.^9^ Hence, the gut microbiota profile is closely tied to the BA pool composition and secondary BA levels, which are associated with various diseases.^8^ For instance, in L2-IL1B mice, treatment with deoxycholic acid (DCA), a DNA-damaging^10^ and carcinogenic secondary BA,^11^^,^^12^ induced substantial aggravation of the phenotype.^13^

Among the BA-activated receptors, the farnesoid X receptor (FXR) is the most important one, as it is the main modulator of BA homeostasis and enterohepatic circulation. Although FXR can bind to many BAs, the downstream effects of FXR vary across BAs. FXR is a nuclear “orphan class” receptor, the main BA receptor, and it is primarily expressed in the small intestine and the liver. Upon ligand-mediated activation, FXR forms a heterodimer with the retinoid X receptor (RXR), binds to specific DNA elements, and regulates the expression of downstream target genes.^14^ FXR activation is important in BA metabolism and homeostasis. Additionally, if activated, FXR was shown to inhibit intestinal tumorigenesis^15^ and hepatic tumor cell proliferation, dedifferentiation, and migration.^16^ It has also been described to inhibit inflammatory signaling.^17^ Moreover, in a colorectal cancer model, FXR expression was downregulated upon HFD exposure, and treatment with an FXR agonist ameliorated the phenotype of these mice.^18^

The manifold functions of FXR intervening in metabolic and tumorigenic signaling make FXR an attractive therapeutic target for treatment of BA-mediated metabolic and gastrointestinal diseases.^19^ In addition, as a first drug targeting FXR, obeticholic acid (OCA), a semisynthetic, highly specific FXR agonist, was approved for treatment of primary biliary cholangitis. In light of the continued rise in GEAC incidence, safe and effective chemopreventive agents could have a significant public health impact.

Here, we analyze the effects of dietary-modulated intestinal microbiota on GEAC carcinogenesis utilizing the L2-IL1B mouse model and assessed the BA signatures of healthy controls and patients with BE and GEAC. We provide evidence of diet-related changes in the intestinal microbiota composition having an impact on BA metaboliam and FXR levels and, hence, on cancer-associated pathways in the metaplastic BE regions in mice and humans. Our results outline a novel concept of how the diet-microbiota-BA axis can affect tumorigenesis.

## Results

### HFD Induces Changes to BA Composition in the Gut, Serum, and BE Tissue

**Figure 1:**
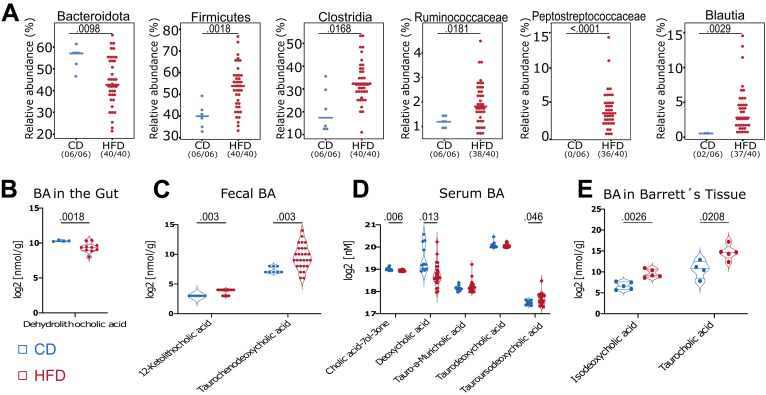
**HFD alters BA metabolism by enrichment of bacteria with BA-converting capabilities leading to a shift in the BA profile in feces, serum, local and distal tissue.***(A)* Relative abundance of bacteria with BA-deconjugating and BA-converting capabilities differs between CD and HFD: Bacteroidota (*P* = .0098); Firmicutes (*P* = .0018); Clostridia (*P* = .0168); Ruminococcacaea (*P* = .0181); Peptostreptococcaceae (*P* < .0001); Blautia (*P* = .0029). *(B)* Among the 42 BAs detected in the gut tissue, dehydrolithocholic acid decreased (*P*_*adj*_ = .0018) in mice fed HFD (n = 10) compared with mice fed CD (n = 4). *(C)* Among the 39 BAs detected in the stool samples, 12-Ketolithocholic acid (*P*_*adj*_ = .003) and TCDCA (*P*_*adj*_ = .003) increased in mice fed HFD (n = 27) compared with mice fed CD (n = 8). *(D)* 33 BAs were detected in serum. While cholic acid-7ol-3-one (*P*_*adj*_ = .006) and DCA (*P*_*adj*_ = .013) decreased in mice fed HFD, TalphaMCA, TDCA, and TUCDA (*P*_*adj*_ = .046) increased in mice fed HFD (n = 24) compared with mice fed CD (n = 11). *(E)* Among the 42 BAs detected in the Barrett’s tissue, iso-DCA (*P*_*adj*_ = .0026) and TCA (*P*_*adj*_ = .0208) increased in mice fed HFD (n = 5) compared with mice fed CD (n = 5). For *(A)*, fecal samples of 3- to 12-month-old L2-IL1B mice were combined. Data are presented as mean with SD. For BE, samples of 6- to 9-month-old L2-IL1B mice were combined.

We previously demonstrated that HFD induced alterations to the gut microbiota and accelerated the dysplastic phenotype in L2-IL1B mice.^6^ Here, we found that mice fed HFD had decreased relative abundance of Bacteroidota, a phyla containing taxa with BA-deconjugating capacities,^20^ and a significant increase in bacteria with BA-converting capabilities^20^, ^21^, ^22^ (Figure 1*A*). Moreover, diet shifted the concentration of certain BAs in gut tissue, feces, serum, and BE tissue (Figure 1*B–E*). Interestingly, in BE tissue of the mice fed HFD, levels of isodeoxycholic acid (Iso-DCA) and taurocholic acid (TCA) were significantly increased. Although TCA has been described to promote growth in vitro, DCA has been linked to esophageal carcinogenesis by inducing pathways of inflammation and DNA damage.^23^ TCA is a conjugated form of cholic acid (CA); high levels of circulating cholic acid in humans with BE were associated with high grade dysplasia and esophageal adenocarcinoma (EAC), and also BE tissue gene expression changes including increased DNA replication.^24^ Taken together, these data support the concept that HFD promotes GEAC in part by changing the gut and systemic BA pool, with these BAs ultimately reaching and acting upon GEJ tissue.

### Loss of FXR Aggravates Dysplasia

**Figure 2:**
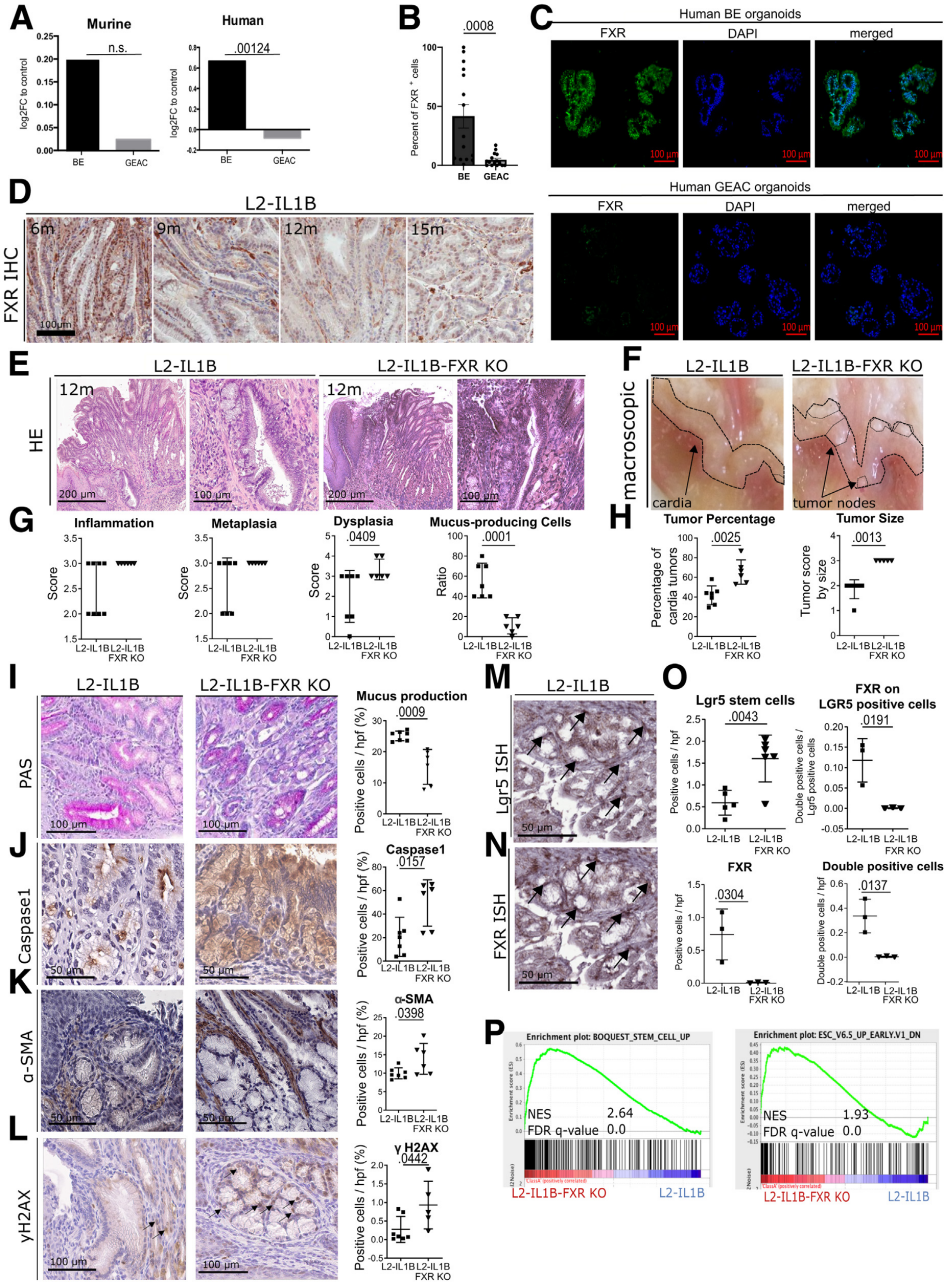
**FXR has a protective role in the malignant progression of BE to GEAC.***(A)* Murine FXR expression data of RNA microarray (GSE24931) in adult BE mice and GEAC mice. Compared with normal tissue, expression of FXR in BE mice was higher and in GEAC mice lower. Analysis of publicly available gene expression data^25^ for FXR in human normal, BE or GEAC tissue samples (GSE13898). Expression of FXR in human BE tissue is significantly higher compared with GEAC tissue (*P*_*adj*_ = .00124). *(B)* Percentage of FXR positive cells in FFPE human organoids of BE (n_organoids_ = 15, n_organoid lines_ = 3) and GEAC (n_organoids_ = 15, n_organoid l__ines_ = 3) patients. Number of FXR-positive cells in BE organoids is significantly higher than in GEAC organoids (*P* = .0008). *(C)* Representative images of FXR immunofluorescence staining (IF) of *(B)*. *(D)* Representative images of FXR IHC at the GEJ of L2-IL1B mice (6-, 9-, 12- and 15-months). *(E)* Representative images of HE staining, *(F)* Representative macroscopic images. *(G)* Although the dysplasia score of L2-IL1B-FXR KO mice was significantly higher (*P* = .0409) than in L2-IL1B mice, the ratio of mucus-producing cells was significantly lower (*P* = .0001). *(H)* Tumor percentage (*P* = .0025) and tumor size (*P* = .0013) were both significantly higher in L2-IL1B compared with L2-IL1B-FXR KO mice. *(I)* Representative images of PAS staining. L2-IL1B-FXR KO mice showed lower (*P* = .0009) mucus production than L2-IL1B mice. *(J)* Representative images of Caspase1 IHC. L2-IL1B-FXR KO mice showed a higher rate (*P* = .0157) of Caspase1 than L2-IL1B mice. *(K)* Representative images of α-SMA IHC. L2-IL1B-FXR KO mice showed a higher rate (*P* = .0398) of α-SMA than L2-IL1B mice. *(L)* Representative images of yH2AX IHC. L2-IL1B-FXR KO mice showed higher DNA damage rate (*P* = .0442) than L2-IL1B mice. *(M)* Lgr5 ISH. *(N)* FXR ISH. *(O)* Compared with L2-IL1B, L2-IL1B-FXR KO mice have an increased (*P* = .0043) number of Lgr5-positive cells, a reduced (*P* = .0304) number of FXR-positive cells, a reduced (*P* = .0191) FXR expression on LGR5 stem cells, and a reduced number of FXR-Lgr5 double positive cells (*P* = .00137). *(P)* Representative gene sets for a stem-cell like phenotype were enriched in a microarray analysis of 12-month L2-IL1B-FXR KO compared with L2-IL1B mice (GSEA of cardia tissue microarray; n = 3). Data are presented as mean with SD. *(G–O)* Data are displayed as positive cells/hpf = area BE region (μM^2^)/1000; In total, 12-month old L2-IL1B (6 females and 5 males) and 12-month old L2-IL1B-FXR KO (5 females and 4 males) were used for this part of the study. *(G–L)* n_L2-IL1B_ = 7 and n_L2-IL1B-FXR KO_ = 6. *(O)* n_L2-IL1B_ = 3–6; n_L2-IL1B-FXR KO_ = 3–6. *(P)* Gene expression data was extracted from microarray analysis, and GSEA was performed using the Broad Institute GSEA software.

**Figure 3:**
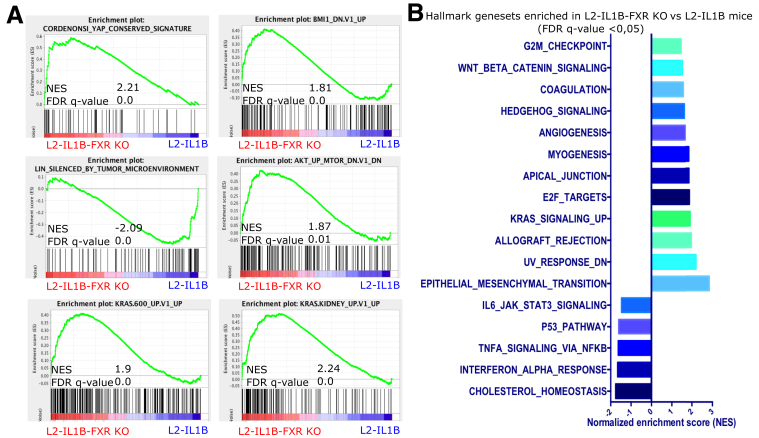
**Microarray analysis shows enrichment of pro-tumorigenic pathways and genes in L2-IL1B FXR-KO compared with L2-IL1B littermates.***(A)* GSEA showed enrichment of YAP, BMI1, AKT, and KRAS signaling pathways, as well as the increased epigenetically induced silencing of the CST6 tumor suppressor gene in L2-IL1B-FXR KO compared with L2-IL1B mice (GSEA of cardia tissue microarray; n = 3). *(B)* Significantly enriched hallmark gene sets in L2-IL1B-FXR KO compared with L2-IL1B mice (FDR q-value ≤0.05).

An important modulator in BA metabolism is FXR. Analysis of previous mRNA expression data of human and mouse^13^ demonstrates that FXR is upregulated in BE but not in GEAC (Figure 2*A*). This was confirmed by immunofluorescence of human BE and GEAC organoids when stained for FXR (Figure 2*B–C*) and by immunohistochemstry in the GEJ of L2-IL-1B mice (Figure 2*D*). For disease evaluation, several parameters, including tumor coverage, dysplasia score, and mucus production, were considered. Compared with L2-IL1B mice, L2-IL1B mice with whole body FXR knockout (L2-IL1B-FXR KO) had increased inflammation, dysplasia, and tumor formation with associated decreased differentiation reflected by the reduction of goblet cells (Figure 2*E–H*). Abrogation of FXR signaling correlated with reduced periodic acid-Schiff (PAS)-positive mucus-producing cells, increased caspase 1 inflammasome activation, α-smooth muscle actin (SMA)-positive fibroblast infiltration, DNA damage, and increased numbers of Lgr5 progenitor cells at the GEJ (Figure 2*I–O*). Importantly, we observed co-localization of Lgr5 and FXR in columnar cells at the GEJ in L2-IL1B mice and an upregulation of stem cell signaling pathways upon loss of FXR, pointing to a direct impact of FXR on Lgr5+ progenitor cells (Figure 2*M–P*). Transcriptome comparisons between L2-IL1B and L2-IL1B-FXR KO mice suggested that loss of FXR promoted tissue reorganization and pro-tumorigenic signaling pathways, such as K-RAS and P53 (Figure 3*A–B*). These data suggest that FXR has a protective effect in BE tissue, and loss of its expression, specifically on progenitor cells, correlates with de-differentiation and disease progression to dysplasia.

### Treatment With FXR Agonist OCA Ameliorates Phenotype In Vitro and In Vivo

**Figure 4:**
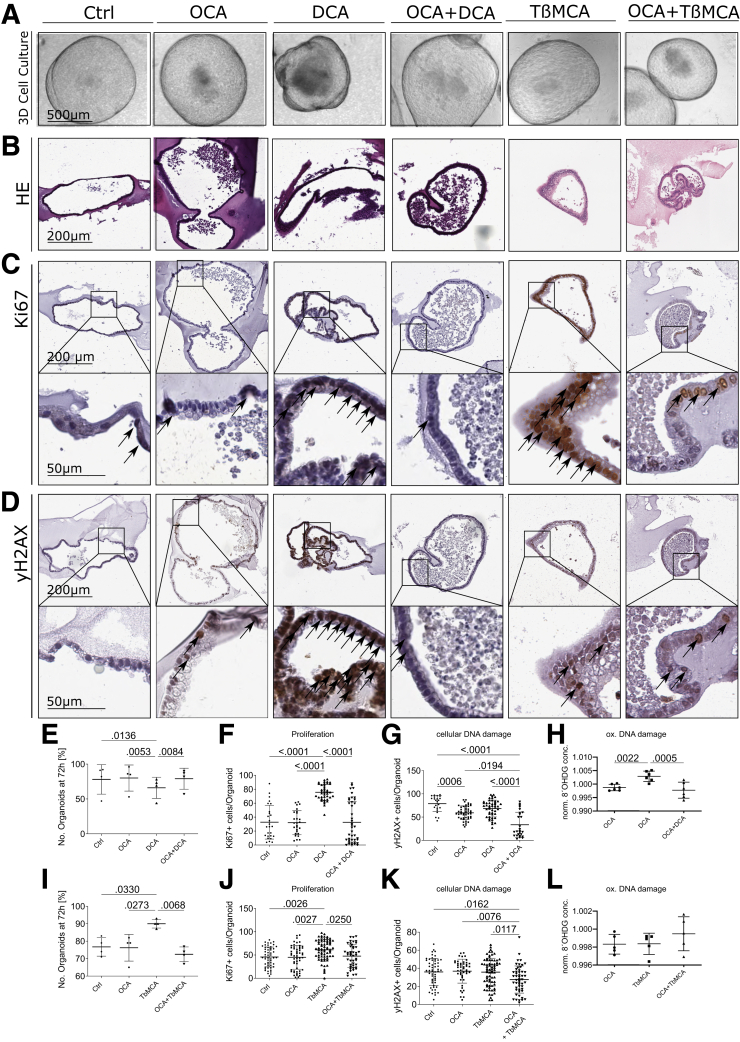
**FXR agonist OCA rescues the damaging and proliferative effects of BAs on organoids of L2-IL1B mice.** Organoids were treated with 10 μM OCA, DCA, OCA + DCA, TßMCA, or OCA + TßMCA in the media for 72 hours, respectively. Organoids were evaluated at 0 hours and 72 hours. *(A)* Representative images of organoid cultures. Compared with all other treatments, DCA-treated organoids showed irregular morphologies and an accumulation of dead cells in the center. *(B)* Representative images of HE staining showing the organoid histology. *(C)* Representative images of Ki67 IHC on organoids. *(D)* Representative images of yH2AX IHC on organoids. *(E)* Organoids treated with DCA showed the lowest numbers compared with control (*P*_*adj*_ = .0136), OCA- (*P*_*adj*_ = .0053), and OCA + DCA- (*P*_*adj*_ = .0084) treated organoids. *(F)* Organoids treated with DCA show the highest proliferation rate compared with control (*P*_*adj*_ < .0001), OCA- (*P*_*adj*_ < .0001), and OCA + DCA- (*P*_*adj*_ < .0001) treated organoids. *(G)* Additional treatment with OCA decreases DNA damage caused by DCA (*P*_*adj*_ < .0001). *(H)* DCA-treated organoids show the highest levels of oxidative DNA damage compared with OCA- (*P*_*adj*_ = .0022) and OCA + DCA- (*P*_*adj*_ = .0005) treated organoids. *(I)* Organoids treated with TβMCA showed the highest numbers compared with control (*P*_*adj*_ = .0330), OCA- (*P*_*adj*_ = .0273) and OCA + TβMCA- (*P*_*adj*_ = .0068) treated organoids. *(J)* Organoids treated with TβMCA showed the highest proliferation rate compared with control (*P*_*adj*_ = .0026), OCA- (*P*_*adj*_ = .0027) and OCA + TβMCA- (*P*_*adj*_ = .0250) treated organoids. *(K)* Additional treatment with OCA decreases DNA damage caused by TβMCA (*P*_*adj*_ = .0117). *(L)* There was no difference between the treatment groups. *(E–L)* Data are presented as mean with SD. *(E, I)* Number of organoids [%] after 72-hour treatment in relation to timepoint 0. Each dot represents a well containing organoids from a single mouse (n_E_ = 5, n_I_ = 4); *(F, J)* Proliferation assessment via Ki67-IHC. *(G, K)* Assessment of DNA damage via yH2AX IHC. Each dot represents the percentage of positive cells per organoid (n_F_ = 26–41; n_G_ = 23–48; n_J_ = 45–69; n_K_ = 46–71) with organoids from 3 different mice combined. *(J, K)* Assessment of proliferation and DNA damage was also performed on organoids treated with 100 μM. Results are not shown but are very similar to those of the treatment with 10 μM. *(H, L)* Assessment of oxidative DNA damage by measuring the 8′OHDG in conditioned media via ELISA. Each dot represent the condition media of a well containing organoids from a single mouse (n_H_ = 6; n_L_= 5). Here, OCA-, DCA-, OCA + DCA-, TβMCA and OCA + TβMCA-treated organoids were normalized to controls.

**Figure 5:**
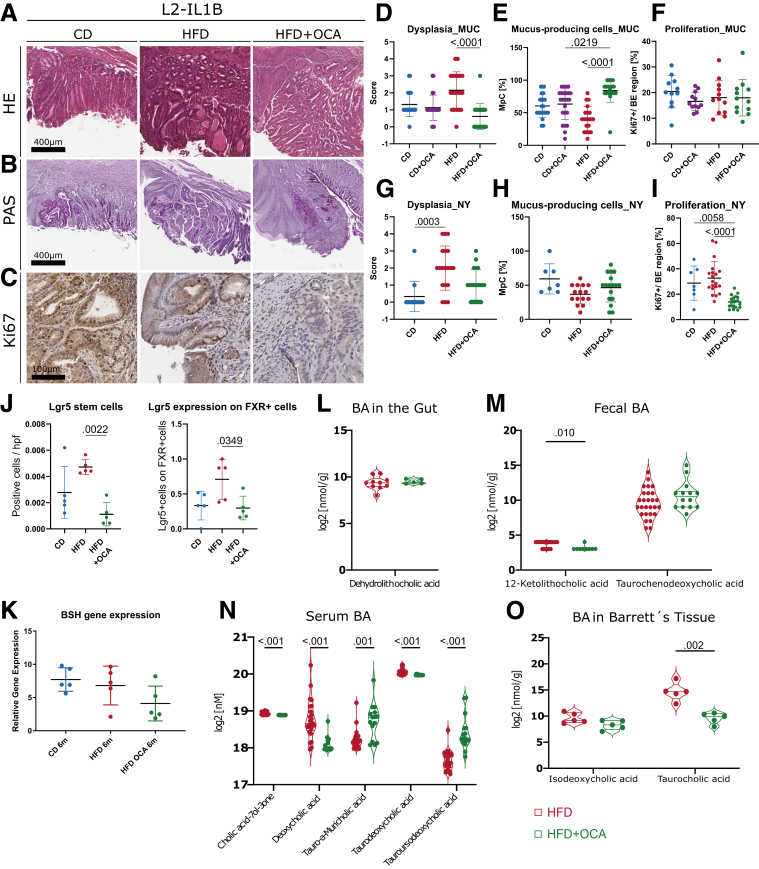
**OCA ameliorates the dysplastic phenotype of HFD-fed L2-IL-1B mice and alters the BA profile in feces, serum, local and tissue.***(A)* Representative images of HE staining. *(B)* Representative images of PAS staining. *(C)* Representative images of Ki67 staining. *(D)* Mice fed HFD + OCA (n = 18) had decreased dysplasia compared with HFD- (n = 20) fed mice (*P*_*adj*_ < .0001); CD (n = 22), CD + OCA (n = 24). *(E)* Mice fed HFD + OCA (n = 18) had an elevated ratio of mucus-producing cells compared with CD + OCA- (n = 24; *P*_*adj*_ = .0219) and HFD- (n = 20; *P*_*adj*_ < .0001) fed mice; CD (n = 22). *(F)* Proliferation of cells at the GEJ was similar in all cohorts; CD (n = 11), CD + OCA (n = 12), HFD (n = 12), HFD + OCA (n = 12). *(G)* Mice fed HFD (n = 19) showed increased dysplasia compared with CD- (n = 12) fed mice (*P*_*adj*_ = .0003); HFD + OCA (n = 20). *(H)* Ratio of mucus-producing cells did not differ between the groups; CD (n = 7), HFD (n = 15), HFD + OCA (n = 17). *(I)* Mice fed HFD + OCA (n = 20) showed lower proliferation of cells at the GEJ than CD- (n = 7; *P*_*adj*_ = .0058) and HFD- (n = 20; *P*_*adj*_ < .0001) fed mice. *(J)* Lgr5 positive cells (*P*_*adj*_ = .0058) and Lgr5 expression on FXR positive cells *P*_*adj*_ = .0058) in HFD- (n = 5) fed mice is significantly increased compared with HFD + OCA- (n = 5) fed mice; CD (n = 5). Data are displayed as positive cells/high power field (hpf) = area GEJ (μm^2^)/1000. *(K)* Gene expression of BSH did not differ between the groups; CD (n = 4), HFD (n = 5), HFD + OCA (n = 5). *(L)* 42 BAs were detected in the intestinal tissue. Concentration of dehydrolithocholic acid did not differ between mice fed HFD (n = 10) and mice fed HFD + OCA (n = 5). *(M)* Among the 39 BAs detected in the stool samples, 12-ketolithocholic acid decreased (*P*_*adj*_ = .010) in mice fed HFD + OCA (n = 14) compared with mice fed HFD (n = 27). Concentration of TCDCA did not differ between mice fed HFD and HFD + OCA. *(N)* 33 BAs were detected in serum. Althouh Cholic acid-7ol-3-one (*P*_*adj*_ < .001), DCA (*P*_*adj*_ < .001) and TDCA (*P*_*adj*_ < .001) decreased in mice fed HFD + OCA, tauro-alpha-muricholic acid (*P*_*adj*_ = .001) and TUDCA (*P*_*adj*_ < .001) increased in mice fed HFD + OCA (n = 15) compared with mice fed HFD (n = 24). *(O)* Among the 42 BAs detected in the Barrett’s tissue, TCA decreased (*P*_*adj*_ = .002) in mice fed HFD + OCA (n = 5) compared with mice fed HFD (n = 5). Concentration of iso-DCA did not differ between mice fed HFD and HFD + OCA. *(D–I)* 6- to 9-month-old L2-IL1B mice from the Munich *(D–F)* or the New York *(G–K)* cohort. Gender distribution of all mice from the Munich cohort: CD (11 females 14 males), CD + OCA (10 females and 16 males), HFD (10 females and 14 males), HFD + OCA (12 females and 11 males). Gender distribution of all mice from the NY cohort: CD (8 females and 3 males), HFD (9 females and 13 males); HFD + OCA (10 females and 5 males). *(F–G)* At this point, it is worth mentioning, that pL2-IL1B-FXR KO mice did not tolerate HFD and died a few weeks after dietary intervention.

**Figure 6:**
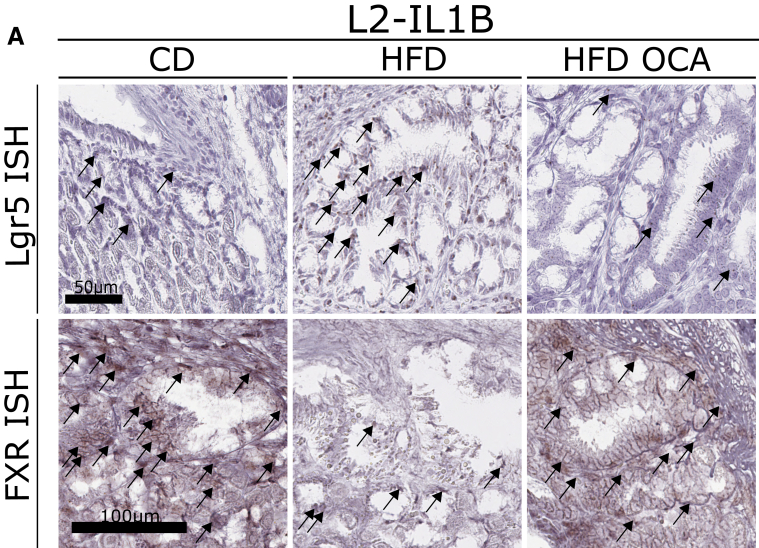
**Lgr5 and FXR expression in L2-IL1B mice treated with CD, HFD, and HFD + OCA.***(A)* Representative pictures of Lgr5- and FXR- ISH of 9-month-old L2-IL1B mice treated with CD, HFD, or HFD + OCA.

**Figure 7:**
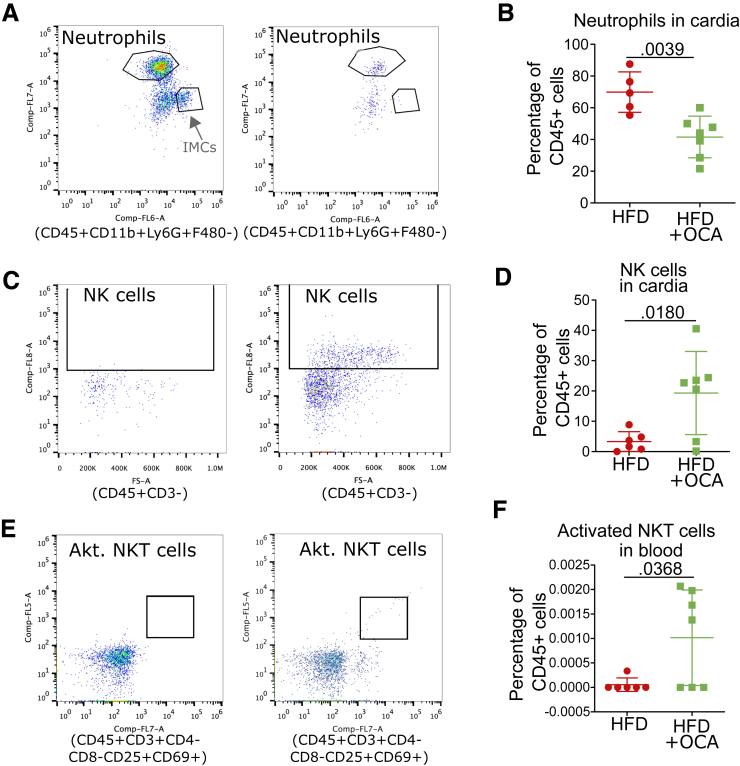
**HFD OCA mice display altered numbers of neutrophils, NKs, and activated NKT cells.***(A)* Representative gating schemes of neutrophils in the cardia of L2-IL1B HFD-fed and HFD + OCA-fed mice. *(B)* The percentage of neutrophils in the cardia region of HFD + OCA-fed compared with HFD-fed mice significantly decreased (n = 6–7l *P*_*adj*_ = .0039). *(C)* Representative gating schemes of NK cells in the cardia of L2-IL1B HFD-fed and HFD + OCA-fed mice. *(D)* The percentage of NK cells in the cardia region of HFD + OCA-fed compared with HFD-fed mice significantly increased (n = 6–7; *P*_*adj*_ = .0180). *(E)* Representative gating schemes of NKT cells in the blood of L2-IL1B HFD-fed and HFD + OCA-fed mice. *(F)* The percentage of activated NKT cells in the blood of HFD + OCA-fed compared with HFD-fed mice significantly increased (n = 6–7; *P*_*adj*_ = .0368). For *(B, D, F)* data are presented as mean percentage of CD45+ cells with SD. For statistical evaluation, unpaired, 2-tailed *t*-tests were performed.

To better understand the effects of FXR, we treated BE organoids isolated from L2-IL1B mice with tauro-beta-muricholic acid (TβMCA), a BA known to inhibit FXR^18^^,^^25^, ^26^, ^27^, ^28^ and with DCA, a BA known to interact with FXR and to be associated with dysplasia and cancer.^13^^,^^18^^,^^29^^,^^30^ We combined this treatment with or without the selective FXR agonist OCA. DCA treatment led to lower organoid numbers despite increased proliferation. DCA also led to increased cellular and oxidative DNA damage (Figure 4*A–H*). TβMCA treatment did not increase cellular and oxidatige DNA damage but increased proliferation and organoid numbers (Figure 4*A–D, I–L*). As suspected, OCA prevented the toxic and proliferative effects of both secondary BAs (Figure 4*A–L*). To examine the effect of OCA on disease progression *in vivo*, we treated HFD-fed mice with OCA and compared them with matched control diet (CD)- and HFD-fed mice. Considering that the microbiota potentially play a role in the manifestation of the phenotype, we performed this *in vivo* study in two locations: Munich (MUC) and New York (NY). The MUC cohort showed decreased dysplasia, increased ratio of mucus-producing cells, and no difference in proliferating cells at the GEJ of mice fed HFD + OCA compared with HFD-fed mice (Figure 5*A-F*). The fact that OCA did not have any impact if combined with CD, presumably due to reduced BA (Figure 1*B-E*), led to the replication of the study without inclusion of CD + OCA in another mouse facility. The NY cohort showed similar but not significant trends and a significant reduction of proliferation in mice fed HFD + OCA compared with CD and HFD (Figure 5*A-C, G-I*). HFD also induced an increase in Lgr5+ and Lgr5+/FXR+ cells at the GEJ (Figure 5*J*; Figure 6). HFD-fed mice treated with OCA treatment also led to reduced levels of neutrophils in BE tissue (Figure 7*A–B*) and elevated numbers of natural killer (NK) and activated natural killer T (NKT) cells (Figure 7*C–F*). Consequently, we showed *in vitro* and *in vivo*, that the FXR agonist OCA can prevent the acceleration of dysplasia, likely due to its effects on the progenitor cell population at the GEJ.

### OCA Treatment Reduces the Content of Growth-promotining Secondary BA

**Figure 8:**
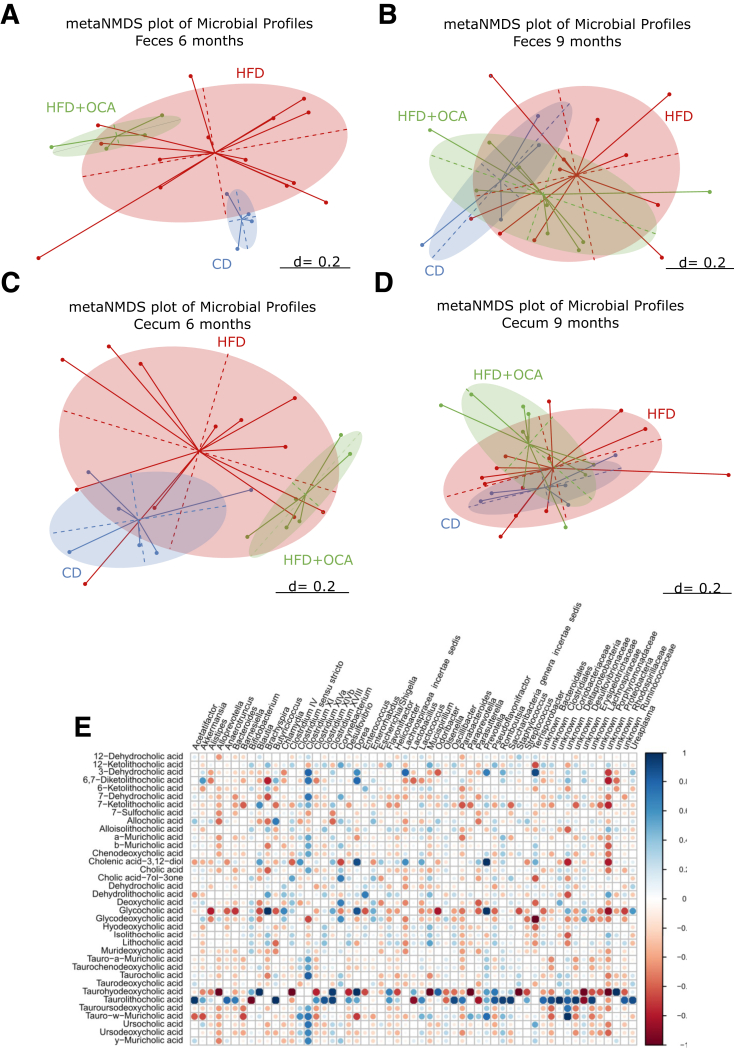
**Beta diversity of fecal and cecal microbial profiles and correlation analysis between fecal BA levels and microbial genera in L2-IL1B mice.***(A)* Meta–Normalized Multidimensional Scaling (metaNMDS) plot of feces from 6-month-old L2-IL1B mice fed with CD, HFD, or HFD + OCA showed significant separation of study groups in terms of bacterial community composition (*P* = .002). *(B)* metaNMDS plot of feces from 9-month-old L2-IL1B mice fed with CD, HFD, or HFD + OCA showed no significant separation of study groups in terms of bacterial community composition (*P* = .214). *(C)* metaNMDS plot of cecum from 6-month-old L2-IL1B mice fed with CD, HFD, or HFD + OCA showed significant separation in terms of bacterial community composition (*P* = .002). *(D)* metaNMDS plot of cecum from 9-month-old L2-IL1B mice fed with CD, HFD, or HFD + OCA showed no significant separation of study groups in terms of bacterial community composition (*P* = .289). *(E)* Correlation analysis between fecal BA levels and the abundance of fecal microbial genera in L2-IL1B mice fed with CD, HFD, or HFD + OCA. For *(A–D)* Permutational Multivariate Analysis of Variance (PERMANOVA) test was used for statistical comparison. The microbiota phylogenetic distances were evaluated through the generalized UniFrac distance. Each point represents the microbiota composition of one sample. *(E)* Correlation analyses were performed using the Rhea pipeline for 16S rRNA-sequencing data.

**Figure 9:**
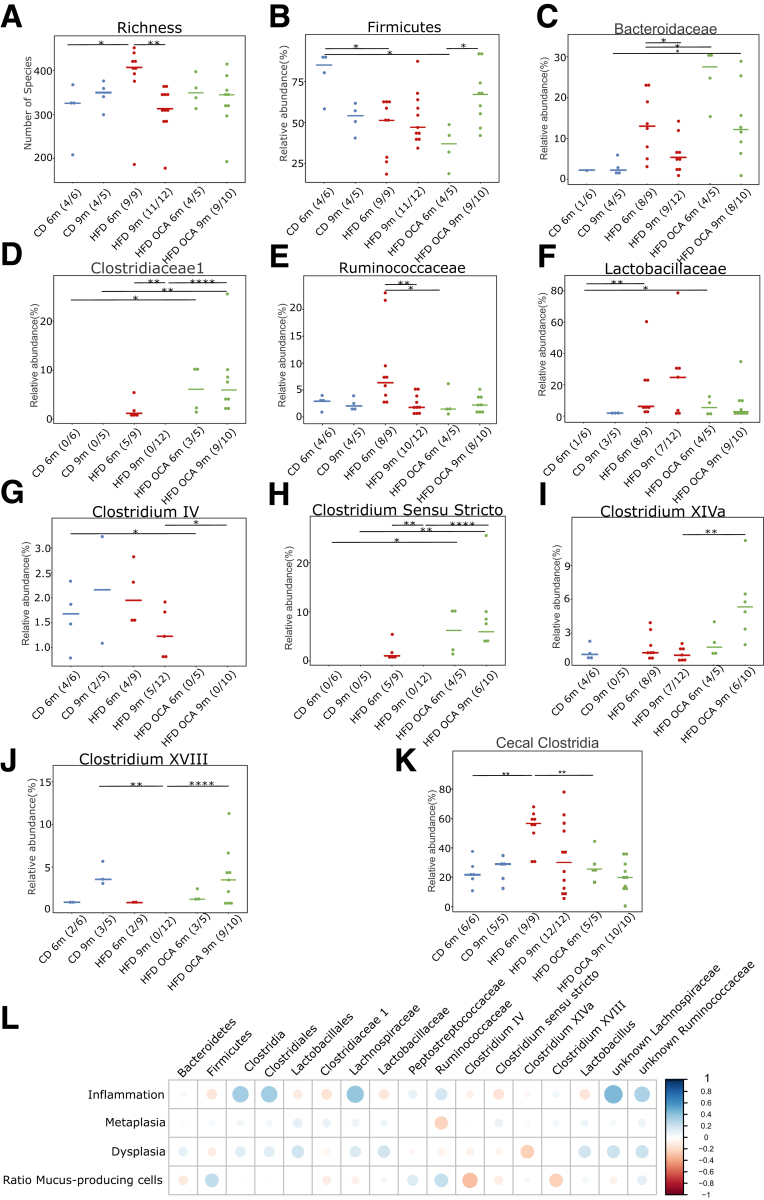
**Treatment with OCA changes the BA-metabolizing gut microbiome in L2-IL1B mice.** Only relevant significant changes are shown. *(A)* Bacterial community richness increased in the feces of 6-month-old HFD-fed mice compared with CD-fed mice (*P* = .0336). Although the richness in both CD and HFD + OCA diets remained constant between 6 months and 9 months of age, richness significantly decreased within the HFD cohort (HFD 6 months vs HFD 9 months; *P* = .0016). *(B)* The relative abundance of the phylum Firmicutes decreased in both 6-month-old HFD-fed (*P* = .0336) and HFD + OCA-fed (*P* = .0286) mice compared with CD-fed mice and increased in 9-month-old compared with 6-month-old HFD + OCA-fed mice (*P* = .0196). *(C)* The relative abundance of the family Bacteroidaceae significantly increased in 6-month-old HFD + OCA-fed compared with HFD-fed and in 9-month-old HFD + OCA-fed compared with CD-fed mice (CD 9 months vs HFD + OCA 9 months; *P* = .0485; HFD 6 months vs HFD + OCA 6 months; *P* = .0283), whereas the abundance decreased in HFD-fed mice from 6 to 9 months of age (HFD 6 months vs HFD 9 months; *P* = .0464). *(D)* The family *Clostridiaceae 1* were only abundant in 6-month-old HFD-fed and 6-month- and 9-month-old HFD + OCA-fed mice (CD 6 months vs HFD + OCA 6 months; *P* = .0286; CD 9 months vs HFD + OCA 9 months; *P* = .0070; HFD 9 months vs HFD + OCA 9 months; *P* = .0001; HFD 6 months vs HFD 9 months; *P* = .0081). *(E)* The relative abundance of the family of *Ruminococcaceae* decreased in both 9-month-old HFD-fed and 6-month-old HFD + OCA-fed mice compared with 6-month-old HFD-fed mice (HFD 6 months vs HFD 9 months; *P* = .0044; HFD 6 months vs HFD + OCA 6 months; *P* = .0485). *(F)* The family of *Lactobacillaceae* was significantly higher abundant in 6-month-old HFD-fed and HFD + OCA-fed compared with 6-month-old CD-fed mice (CD 6 months vs HFD 6 months; *P* = .0070; CD 6 months vs HFD + OCA 6 months; *P* = .0286). *(G)* The genus *Clostridium* cluster IV was only abundant in CD- and HFD-fed mice (CD 6 months vs HFD + OCA 6 months; *P* = .0286; HFD 9 months vs HFD + OCA 9 months; *P* = .0379). *(H)* The genus *Clostridium sensu stricto* was significantly higher abundant compared with 6-month- and 9-month-old CD-fed mice and compared with 9-month-old HFD-fed mice (CD 6 months vs HFD + OCA 6 months; *P* = .0286; CD 9 months vs HFD + OCA 9 months; *P* = .0070; HFD 9 months vs HFD + OCA 9 months; *P* = .0001). Abundance dropped in HFD-fed mice from 6 to 9 months of age (*P* = .0081). *(I)* The *Clostridium cluster XIV* was significantly more abundant in 9-month-old HFD + OCA-fed mice compared with HFD-fed mice (*P* = .0023). *(J)* The genus *Clostridium XVIII* was only abundant in 6-month-old HFD-fed and in 6-month- and 9-month-old HFD + OCA-fed mice (CD 9 months vs HFD 9 months; *P* = .0088; HFD 9 months vs HFD + OCA 9 months; *P* < .0001). *(K)* In the cecal content of the mice, the class of Clostridia was significantly enriched in abundance in 6-month-old HFD-fed compared with CD- and HFD + OCA-fed mice (HFD 6 months vs CD 6 months; *P* = .0016; HFD 6 months vs HFD + OCA 6 months; *P* = .0040). *(L)* Correlation analysis between the abundance of bacterial families with BA metabolizing capacities in all treatment groups vs inflammation, metaplasia, and dysplasia scores as well as the ratio of mucus-producing cells. Correlation analyses were performed using the Rhea pipeline for 16s-sequencing data. Clostridia, Lactobacillales, and *Lachnospiraceae* and unknown *Ruminococcaceae* were the main microbial groups positively correlated with dysplasia scores. For *(A–K)*. relative abundances of microbiota are represented as mean. For statistical analysis, pairwise Wilcoxon rank sum test or Fisher’s exact test, if appropriate, were used.

**Figure 10:**
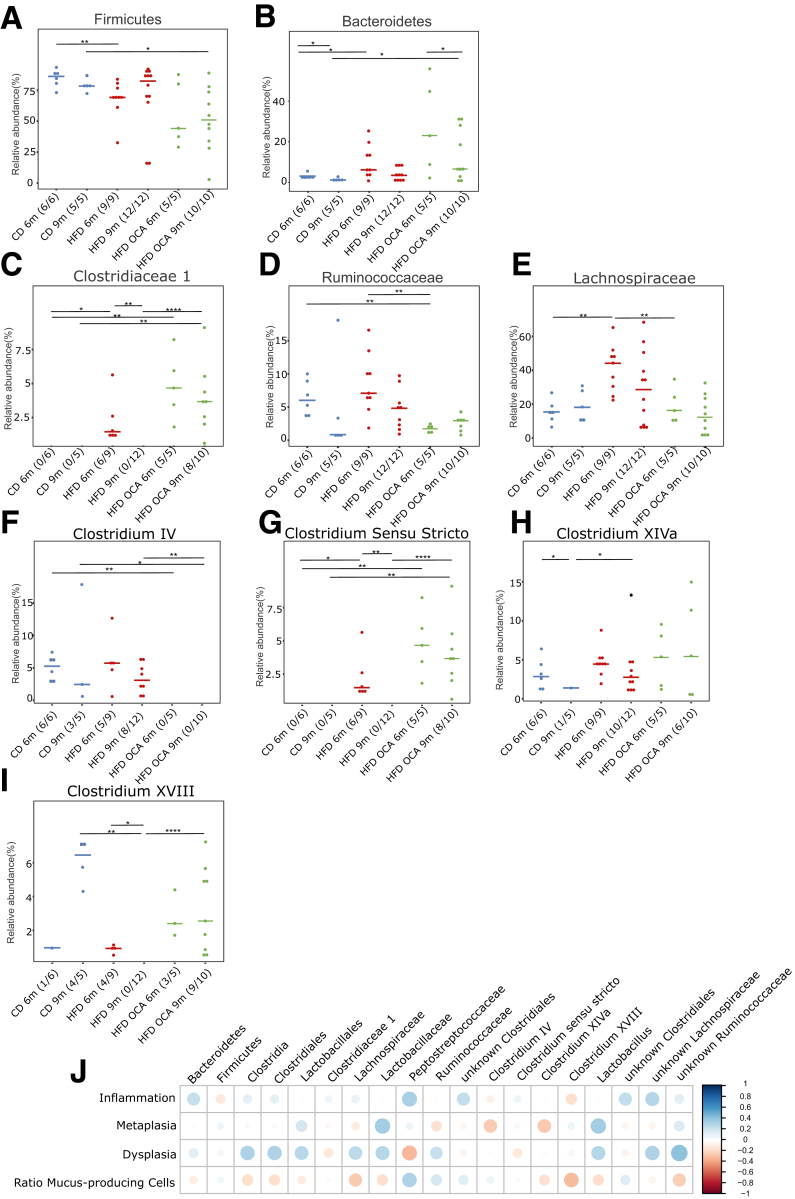
**Representation of the cecal relative abundance (%) of bacterial families with BA metabolizing capacities between L2-IL1B mice fed with CD, HFD or HFD+OCA at 6 month and 9 month of age.** Only relevant significant changes are shown. *(A)* The phylum Firmicute*s* decreased in abundance in 6-month-old HFD- compared with CD-fed mice and in 9-month-old HFD + OCA- compared with CD-fed mice (CD 6 months vs HFD 6 months; *P* = .0076; CD 9 months vs HFD + OCA 9 months; *P* = .0400). *(B)* The phylum Bacteroides was significantly higher abundant in 6-month-old HFD- and HFD + OCA-fed compared with CD-fed mice. The relative abundance dropped between 6-month- and 9-month-old in CD- and HFD + OCA-fed mice (CD 6 months vs CD 9 months; *P* = .0303; CD 6 months vs HFD 6 months; *P* = .0256; CD 9 months vs HFD + OCA 9 months; *P* = .0400; HFD + OCA 6 months vs HFD + OCA 9 months; *P* = .0400). *(C)* The family *Clostridiaceae 1* were only abundant in 6-month-old HFD-fed and 6-month- and 9-month-old HFD + OCA-fed mice (CD 6 months vs HFD 6 months; *P* = .0278; CD 6 months vs HFD + OCA 6 months; *P* = .0022; CD 9 months vs HFD + OCA 9 months; *P* = .0070; HFD 9 months vs HFD + OCA 9 months; *P* = .0001; HFD 6 months vs HFD 9 months; *P* = .0015). *(D)* The family of *Ruminococcaceae* was significantly decreased in abundance in 6 month-old HFD + OCA-fed mice compared with CD- and HFD-fed mice (CD 6 months vs HFD + OCA 6 months; *P* = .0043; HFD 6 months vs HFD + OCA 6 months; *P* = .0040). *(E)* The family of *Lachnospiraceae* was significantly enriched in abundance in 6-month-old HFD-fed mice compared with CD- and HFD + OCA-fed mice (CD 6 months vs HFD 6 months; *P* = .0016; CD 9 months vs HFD 6 months; *P* = .0120). *(F)* The genus *Clostridium cluster IV* was only abundant in CD- and HFD-fed mice (CD 6 months vs HFD + OCA 6 months; *P* = .0022; CD 9 months vs HFD + OCA 9 months; *P* = .0220; HFD 9 months vs HFD + OCA 9 months; *P* = .0017). *(G)* The genus *Clostridium sensu stricto* was only abundant in 6-month-old HFD-fed and in 6-month- and 9-month-old HFD + OCA-fed mice (CD 6 months vs HFD 6 months; *P* = .0278; CD 6 months vs HFD + OCA 6 months; *P* = .0022; CD 9 months vs HFD + OCA 9 months; *P* = .0070; HFD 9 months vs HFD + OCA 9 months; *P* = .0001; HFD 6 months vs HFD 9 months; *P* = .0015). *(H)* Abundance of the genus *Clostridium cluster XIVa* was increased in 9-month-old HFD- vs CD-fed mice (*P* = .0276) and decreased in 9-month- vs 6-month-old CD-fed mice (*P* = .0152). *(I)* The genus *Clostridium cluster XVIII* was significantly higher abundant in 9-month-old CD- and HFD + OCA-fed compared with HFD-fed mice (CD 9 months vs HFD 9 months; *P* = .0021; HFD 9 months vs HFD + OCA 9 months; *P* < .0001) and was not present in 9-month-old HFD-fed mice (HFD 6 months vs HFD 9 months; *P* = .0211). *(J)* Correlation analysis between the abundance of bacterial families with BA metabolizing capacities in all treatment groups vs inflammation, metaplasia, and dysplasia scores as well as the ratio of mucus-producing cells. Correlation analyses were performed using the Rhea pipeline for 16s-sequencing data. *Clostridia, Lactobacillales, Ruminococcaceae,* and unknown *Lachnospiraceae* were the main microbial groups positively correlated with dysplasia scores. For *(A–J)*, relative abundances of microbiota are represented as mean. For statistical analysis, pairwise Wilcoxon rank sum test or Fisher’s exact test, if appropriate, were used.

Considering that metabolic changes in the gut seem to be linked to systemic metabolic changes, we further delved into the microbiota-metabolome-phenotype axis. Bile salt hydrolase (BSH) is a microbial, BA-deconjugating enzyme that deconjugates primary BAs, which unconjugated, can be then transformed into secondary BAs and be further re-conjugated.^31^ Expression of BSH was reduced by OCA treatment in HFD-fed mice (Figure 5*K*). The fecal bacterial profile, especially of 6-month L2-IL1B mice, changed significantly between HFD-fed and HFD + OCA-fed mice (Figure 8*A–B*). Microbial analyses showed that bacterial community richness dropped significantly in the feces from 6- to 9-month HFD-fed mice (Figure 9*A*). Additionally, depending on the diet and the age of the mice, a bacterial shift was observed (Figure 9*B–J*). The cecal microbial profile closely resembled the fecal microbial profile (Figure 8*C–D*; Figure 10*A–I*). In both feces and cecal contents, bacteria belonging to Clostridia, Lachnospiraceae, and Ruminicoccaaceae correlated with inflammation, dysplasia, and with the BA taurolithocholic acid (TLCA) (Figure 8*E*; Figure 9*L*). TLCA has been described to promote cell growth in cholangiocarcinoma^32^ and to promote epithelial-mesenchymal transition.^33^ A balanced ratio of Firmicutes/Bacteroidetes (F/B) is widely associated with normal intestinal homeostasis, and alterations in this ratio are linked to gut dysbiosis and certain diseases.^34^ Here, although Bacteroidetes correlated positively with dysplasia and negatively with the ratio of mucus-producing cells, Firmicutes correlated negatively with dysplasia and positively with the ratio of mucus-producing cells (Figure 9*L*).

OCA treatment altered the concentration of certain BAs in gut tissue, feces, and serum of L2-IL1B mice fed with HFD (Figure 5*L–O*). Specifically, OCA treatment decreased serum levels of DCA and taurodeoxycholic acid (TDCA) (Figure 5*N*), which have been described to promote progression of GEAC.^23^ OCA treatment also lowered TCA in BE tissue (Figure 5*O*), which has been linked to invasive growth *in vitro*.^35^ In the serum, OCA treatment additionally increased the concentration of the FXR-antagonist tauro-alpha-turicholic acid (TalphaMCA)^25^ and of tauroursodeoxycholic acid (TUDCA), a BA with anti-inflammatory and anti-carcinogenic properties^36^^,^^37^ (Figure 5*N*). Although being contradictory, the increase of TalphaMCA matches with the high abundance of Clostridia members in feces and cecum of mice fed HFD + OCA (Figure 9*D, G–K* and Figure 10*C, F–I*), an observation that has been described by others^22^ as well. Besides linking TLCA and certain bacteria to inflammation and disease, our data suggests that OCA is capable of reducing contents of growth-promoting secondary BAs such as DCA and TDCA, presumably by decreasing the expression of BSH.

### Stool and Serum BA Profiles in Patients With BE and GEAC are Linked to the Disease Stage

**Figure 11:**
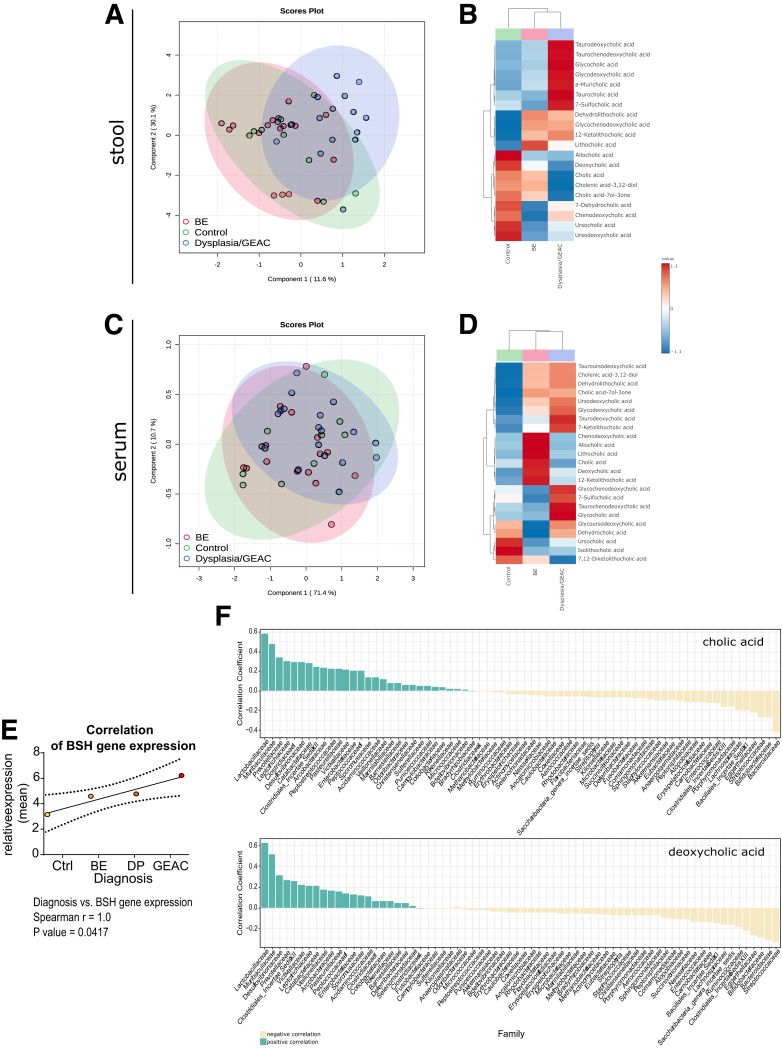
**BA profile in stool and serum is linked to the disease stage in humans.***(A)* PLS-DA of BA profiles in the stool of healthy controls (n = 9), patients with BE (n = 18), and patients with dysplasia/GEAC (n = 16). The BA profile of dysplastic and GEAC patients clustered differently than the healthy controls. *(B)* Clustering of the mean stool BA levels in healthy controls, BE, and dysplasia/GEAC patients shown as heatmap (Euclidean distance measure, ward clustering algorithm). *(C)* PLS-DA of BA profiles in the sera of healthy controls (n = 10), patients with BE (n = 16) and patients with dysplasia/GEAC (n = 19) showed no clear clusters. *(D)* Clustering of the mean serum BA levels in human control, BE and GEAC patients shown as heatmap (Euclidean distance measure, ward clustering algorithm). *(E)* Correlation of gut bacterial BSH gene expression with the diagnostic stages of human patients. Spearman correlation analysis of the mean relative expression of BSH represented by delta Ct. *(F)* Bacteria families were ranked according to the correlation coefficients, which were calculated using the relative abundance of the bacteria family in all individuals and corresponding circulating CA and DCA levels. Only bacteria families with a correlation coefficient greater than 0.5 were considered. The family Lactobacillaceae correlated with levels of both CA and DCA. *(A–D)* Plots were created with data of targeted BA analysis. *(B, D)* Euclidean distance measure, ward clustering algorithm with Euclidean distance measure was applied for the heatmaps.

**Figure 12:**
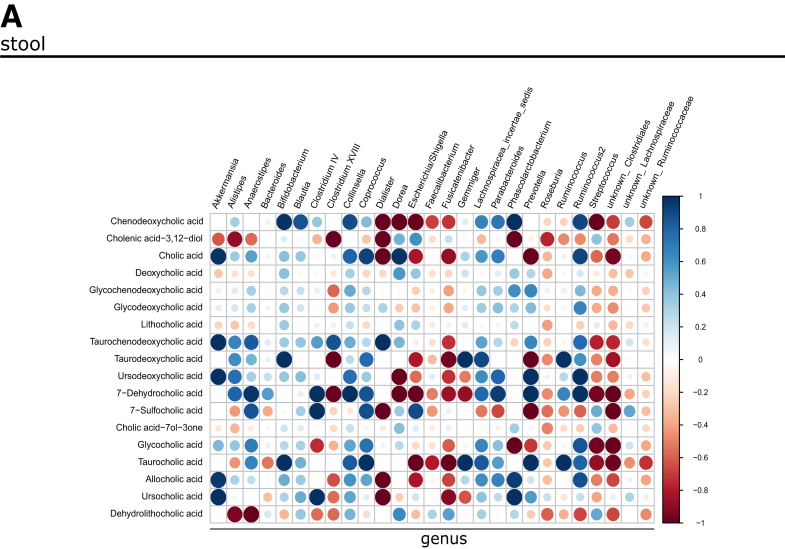
**Intestinal microbial profile at genus level in relation to BA levels in patients.***(A)* Correlation of the relative abundance of bacteria genera in the stool of human patients with disease severity and fecal BA levels. Correlation analyses were performed using the Rhea pipeline for 16S rRNA sequencing data.

To explore the microbiota-metabolome axis at a more clinical level, we analyzed a cohort of patients at different states of esophageal carcinogenesis (non-BE controls, BE, dysplasia, GEAC) from the BarrettNET Registry.^38^ BA profiling of stool showed clustering linked to disease stage, and enriched conjugated BAs in dysplastic patients and patients with GEAC (Figure 11*A–B*). Despite unclear clustering, BA profiling of sera also showed conjugated BAs being enriched in dysplastic patients and patients with GEAC (Figure 11*C–D*). Furthermore, TDCA, taurochenodeoxycholic acid (TCDCA), and glycocholic acid (GCA) were enriched in both stool and serum of patients with dysplastia/GEAC. Although DCA appears to promote carcinogenesis by inducing pathways of inflammation and DNA damage, chenodeoxycholic acid (CDCA) is linked to carcinogenesis by enhancing cell proliferation and promoting of angiogenesis.^23^ Moreover, we identified the bacteria Bifidobacteria, Gremmiger, and Ruminococcus to to strongly correlate with the BAs enriched in the stool of patients with dysplasia/GEAC (Figure 12*A*). Here, we show the expression of BSH to positively correlate with disease severity (Figure 11*E*). In the colon, gut bacteria convert the primary BA CA into the secondary BA DCA.^9^ Interestingly, in patients'sera, levels of these BAs positively correlated with *Lactobacillaceae* (Figure 11*F*), and members of this family have been described to express BSH.^39^ Matching with the hypothesis that microbial metabolites in the gut reach distant tissues^40^ after being absorbed in the gut and entering the circulation, our results show that metabolic changes in the gut can be reflected in the serum.

## Discussion

Here, we hypothesize that in context of GEAC carcinogenesis, loss or inhibition of FXR results in a dysfunctional capacity to compensate for gut-borne BAs, which induce cell stress in progenitor cells at the GEJ.

In L2-IL1B mice fed HFD, we observed an alteration of the gut microbiota with an associated shift in the BA profile of the gut, serum, and BE tissue. The enrichment of cancer-promoting BAs (ie, 12-Keto-LCA, TCDCA, Iso-DCA, and TCA)^23^ in the gut and most importantly, in the BE tissue of HFD-fed mice, suggested these BAs play a role during disease progression. In addition, numerous bacterial groups were negatively associated with inflammation and dysplasia while positively associated with metaplasia, implying that a diet-altered microbiota-metabolome-axis might represent a key factor that shapes the phenotypic fate of the progenitor cells at the GEJ towards differentiated goblet cells or dysplastic cells.^41^^,^^42^

In line with previous studies, we found upregulation of FXR in BE tissue of mice and patients, and downregulation associated with tumor development, suggesting that FXR activation might have a protective effect.^43^^,^^44^ Indeed, genetic deficiency of FXR in L2-IL1B mice promoted and accelerated neoplasia. Loss of FXR led to formation of a tumor-promoting microenvironment, with enrichment of pro-tumorigenic signaling pathways, less protective epithelial mucus production, and increased numbers of Lgr5 progenitor cells. Importantly, co-localization of Lgr5 and FXR in the BE region in L2-IL1B mice and enrichment of stem cell signaling pathways upon loss of FXR pointed to a direct function of FXR in Lgr5+ progenitor cells.

We hypothesize that a drop in FXR expression, together with non-physiologic concentrations of BAs that inhibit FXR at the GEJ, could increase DNA damage and, in turn, promote malignant transformation. Conversely, activation of FXR via its agonist OCA altered the BA pool and showed anti-neoplastic effects. When examining FXR as a therapeutic target, we observed a protective effect of OCA in murine BE organoids by attenuation of cytotoxic effects of the secondary BAs. When translating this into an *in vivo* proof-of principle performed in 2 different facilities, we observed OCA improving the phenotype at the GEJ of HFD-fed mice. Although in the MUC cohort, OCA significantly abrogated dysplasia and promoted differentiation, in the NY cohort, the trend was similar but not significant. Animal facility, breeding, and housing conditions have an effect on the gut microbiota, which plays a role in the manifestation of the phenotype.^45^^,^^46^ This may explain the similar trend but different significance between the 2 cohorts.

Furthermore, the immune profile of OCA-treated HFD-fed mice was altered, with reduced inflammation represented by neutrophil infiltration and elevated numbers of NK and activated NKT cells. Although this observation requires further investigation, it suggests that OCA may have the potential to shift the inflammatory profile in a more anti-tumor direction. In addition, OCA treatment resulted in decreased BSH expression in gut bacteria and reduced abundance of secondary BA producers such as Ruminococcaceae,^47^ pointing to diminished microbial potential for BA transformation in OCA-treated mice.

Although fat metabolism and BA signatures differ between men and mice, complementary to our results in mice, BA levels and some associated microbial alterations in patients with BE correlated with disease progression. However, the limited number of participants of this human cohort encourages more in depth-studies to confirm this clinical concept.

In summary, in a mouse model of BE and GEAC, a diet high in fat modified the composition of the intestinal microbiota and altered BA metabolism and levels in the gut, serum, and distant tissue. Loss or inhibition of FXR was linked with onset of tumorigenesis and resulted in a tumor-promoting microenvironment, characterized by dedifferentiation, progenitor cell expansion, DNA damage, and increased inflammatory responsiveness, facilitating the dysplastic transformation of the epithelial progenitor cells. In mice, treatment with OCA reduced BA levels and ameliorated the dysplastic phenotype. In patients, BA profile in stool and serum correlated with disease progression. We propose a concept in that BAs are metabolized in the gut (colon) and are transported via the blood stream to the esophagus, where they can induce tissue damage and accelerate carcinogenesis in the setting of inflammation (esophagitis). In addition, our data imply that the integrity in expression and function of FXR is crucial to prevent dedifferentiation at the GEJ with subsequent progression into GEAC. Importantly, our results suggest that this acceleration of carcinogenesis can be prevented by treatment with the FXR agonist OCA.

## Materials and Methods

### Transgenic Mouse Strains

The animal experimental work performed in Munich, Germany was carried out under the approval of the district government of Upper Bavaria, according to the animal experimental approvals 55.2.1.54-2532-125-12 and 55.2-1-54-2532-24-2016. The animal experimental work performed in New York, New York, was approved by the Columbia University Institutional Animal Care and Use Committee (AICUC) for breeding, housing, feeding of special diets (CD and HFD), and OCA administration under the identification AC-AAAN5700 (Y3 M03). All animal experiments were conducted in accordance with Animal Welfare and Ethical Guidelines of the Klinikum Rechts der Isar, TUM, Munich and the Columbia University, New York. All animal experimental work in this study was based on the transgenic mouse model (L2-IL1B) of BE and GEAC.^48^ L2-IL1B mice overexpress the human interleukin 1 beta in the esophageal and squamous forestomach mucosa under control of the Epstein-Barr virus promoter (EBV-L2). L2-IL1B mice develop esophagitis with subsequent progression to BE and ultimately to GEAC without any additional intervention. To obtain L2-IL1B-FXR KO mice, L2-IL1B mice were backcrossed to C57BL/6J mice and crossed with FXR knockout (KO) mice, which have a loxP-Cre-based whole-body KO of FXR.^49^

### Dietary Treatment of L2-IL1B Mice

For dietary treatment studies in Germany, starting at 8 to 9 weeks of age, mice were randomly distributed in treatment groups and fed HFD (Ssniff, S5745-E712), a matching CD (Ssniff, S5745-E702), or HFD/CD + obeticholic acid (HFD + OCA, S0615-E710; CD + OCA, Ssniff, S0615-E705). For dietary treatment studies in New York, starting at 8 to 9 weeks of age, mice were randomly distributed in treatment groups and fed CD, HFD, or HFD + OCA (dietary composition analogous to the diets used in Germany).

### Human BarrettNET Study Cohort

A subset of samples and data from the prospective BarrettNET study at Klinikum Rechts der Isar in Munich was used for this work and included samples from patients with non-dysplastic BE (n = 18) and dysplasia or GEAC (n = 19), as well as non-BE controls (n = 10). Stool and serum samples were analyzed for changes in BAs, and stool was analyzed for changes in BA metabolizing bacteria.^40^

### Patient and Public Involvement

Patients were asked to evaluate the process of BarrettNET within a QM questionnaire, and individual study results were communicated to the patient and used for diagnostic and therapeutic evaluation.

### Statistical Analysis

Statistical analyses were performed using GraphPad Prism version 8.00 for Windows (GraphPad Software). Data was presented as mean ± standard deviation (SD) if not stated otherwise. Targeted metabolomic data was presented as mean. Comparison of 2 groups was performed by unpaired *t*-tests. For comparison of more than 2 normally distributed groups, ordinary 1-way analysis of variance (ANOVA) with Tukey's multiple comparison test was performed. If the values were not normally distributed, Mann-Whitney *U* test with Holm-Sidak multiple comparison testing was applied. Histologic scores—being ordinal values—were compared using Kruskal-Wallis tests and Dunn’s post-hoc test.

### Breeding and Mouse Husbandry

For microbiome and metabolome characterization of L2-IL1B mice on CD or HFD, L2-IL1B mice were backcrossed to C57BL/6J mice and bred and maintained under specific pathogen-free (SPF) conditions in the animal facility of the ZIEL Institute for Food and Health at the School of Life Sciences in Weihenstephan (WZW), TUM. L2-IL1B-FXR KO mice were bred and kept under SPF conditions in the animal facility of the Klinikum Rechts der Isar, TUM. For the OCA treatment studies in Germany, L2-IL1B mice were backcrossed to C57BL/6J mice and bred in a mouse facility from Charles River in Italy under SPF conditions. Following weaning and genotyping, mice were sent back to Germany at an age of 6 to 8 weeks. In Germany, all mice were kept under SPF conditions in the animal facility of the Klinikum Rechts der Isar, TUM. For the OCA treatment studies in New York, L2-IL1B mice were backcrossed to C57BL/6J mice and bred and maintained under SPF conditions in an animal facility of the Irving Cancer Research Center, Columbia University, New York.

### Euthanasia, Preparation, Sample Collection, and Disease Evaluation, Including Mucus Assessment at the GEJ

Mice were euthanized by an isoflurane overdose with subsequent cervical dislocation at specific timepoints and adequately dissected; feces, cecal content, blood from cardiac puncture, and organs were subjected to downstream applications, including flow cytometry, formalin-fixed paraffin-embedded (FFPE), and storage at −80 °C for RNA-sequencing, 16S-sequencing, and metabolomic analyses. Images taken from the stomach and esophagus during dissection were evaluated for tumor size and tumor coverage in percent using the software ImageJ.^50^ Macroscopic scores were determined as tumor size score and percentage of tumor coverage at the GEJ (criteria: tumor size score 0 = no abnormalities; 1 = tumors < 0.5 mm; 2 = tumors <1 mm; 3 = tumors <2 mm; 4 = tumors <3 mm). Mouse tissues were fixed in 4% paraformaldehyde and paraffin-embedded; FFPE sections were cut and stained with hematoxylin and eosin (H&E). Histopathologic scores were given by an experienced gastrointestinal pathologist who was blinded to the identity of the specimens. The scoring system followed the previously established criteria for inflammation, metaplasia, dysplasia, and ratio of mucus-producing cells:^13^ Inflammation was scored by estimating the percentage of immune cells infiltrated in the tissue area of the GEJ. Metaplasia score and ratio of mucus-producing cells were estimated based on the abundance of glands with mucus-producing cells in the BE area at the GEJ. Dysplasia was evaluated by the amount of cellular atypia and the presence of low- (LGD) or high-grade dysplasia (HGD) in single or multiple glands. Mucus production was assessed by PAS staining and quantified as percentage of PAS-positive area in BE regions.

### Inclusion Criteria and Sample Collection for the BarrettNET Study

Inclusion criteria in the study included an age between 18 and 80 years, surveillance endoscopy in patients with already diagnosed BE without previously known occurrence of LGD or HGD or GEAC, and no presence of contraindications. Per patient and visit, 4 to 6 endoscopic biopsies from the squamous epithelium of the cardia and BE regions, blood and fecal samples were collected. Biopsies (Preanalytics, PAXgene Tissue Container, Cat 765112) and blood (PAXgene blood DNA tubes 2,5 ml Cat 761165; Sarstedt S-Monovette 5 mL 9NC, Cat. 05.1071) were collected in line with the endoscopy unit at the Klinikum Rechts der Isar, TUM, in Munich, Germany. Fecal samples were collected by the patients (Sarstedt, Stool Collection Tubes with Stool DNA Stabilizer, Cat. 1038111300). Biopsies were processed at the clinical pathology department according to the manufacturer’s protocol. FFPE samples were stored at −20 °C, stabilized blood samples were stored at −80°C or room temperature, and stabilized fecal samples were stored at −80 °C under appropriate storage conditions.

### Flow Cytometry for Analysis of Immune Cells

Single-cell suspensions were generated as previously described.^51^ The following antibodies were used: APC-anti-F4/80, APC-e780-anti-cd11b1β, Alexa700-anti-Ly6G, eFluor450-anti-CD45, PE-Ly6C, eFluor450-anti-CD4, APC-CD8a, FITC-anti-CD3, APCe780-NK1.1, and PE-anti-gamma delta TCR; 7-AAD was used to quantify live cells; all antibodies were purchased from eBioscience. Fluorescence-activating cell sorting (FACS) data were acquired on a Gallios flow cytometer (Beckman Coulter) and analyzed using FlowJo software (FlowJo).

### Immunohistochemistry

Immunohistochemistry (IHC) on sections from FFPE tissue was performed for detection of specific antigens in the tissue. Standard immunohistochemical procedures with citrate buffer antigen retrieval (H-3300, Vector Labs) were conducted with the following antibodies: Ki67 (Abcam, AB15580; 1:1000, 4 °C overnight), αSMA (Abcam, AB5694; 1:400, 4 °C overnight), γH2AX (Cell Signaling, #9718; 1:750, 4 °C overnight), rabbit-anti-mouse Caspase1 (1:50; 30 minutes at room temperature), and TGR5 (Abcam, AB72608; 1:500, 4 °C overnight). Quantification was assessed as the percentage of positive cells in the BE region, which were defined as the region between the squamous epithelium and the oxyntic mucosa of the stomach.

### In Situ Hybridization

In situ hybridization (ISH) was done to detect antigen expression on the mRNA level for Lgr5 and FXR. The RNAscope 2.5 HD assay – Detection reagent BROWN (ACD) and all related reagents from ACD were used. The procedure was performed according to the manufacturer's protocol using the Mm-Lgr5 target probe (ACD, Cat. No. 312171), the Hs-Lgr5 target probe (ACD, Cat. No 311021), and the Mm-NR1H4 target probe (ACD, Cat. No 484491) for detection of FXR expression. Quantification was assessed as the percentage of positive cells in the BE region as for IHC.

### RNA Extraction and Reverse Transcription

Tissue for RNA isolation was collected and stored overnight in 250 μl RNAlater (AM-7020; Invitrogen) at 4 °C before long-term storage at −80°C. Isolation was performed using the RNeasy Mini Kit (74104; Qiagen) according to manufacturer’s instructions. For tissue homogenization, a SilentCrusher M (Heidolph) was employed. RNA was eluted in 20 μL polymerase chain reaction-grade water. RNA concentration and quality were measured on a Nano-Drop 2000 spectrophotometer (Thermo Scientific). RNA was directly subjected to reverse transcription (RT) or stored at -80°C until further use in downstream applications. For RNA extraction from stool, the Quick-RNA Fecal/Soil Microbe Microprep Kit (R2040, Zymo Research) was performed according to manufacturer’s instructions. RT was conducted with the QuantiTect Reverse Transcription Kit (205314, Qiagen) according to manufacturer's instructions.

### Quantitative Real-time Polymerase Chain Reaction

Target gene expression levels were evaluated by quantitative real-time polymerase chain reaction (qRT-PCR) on a LightCycler 480 (Roche). PCR reactions were performed in a total volume of 10 μL per reaction using the QuantiFast SYBR Green PCR Kit (4000) (204057, Qiagen). For each reaction, 10 to 25 ng RNA were applied in a volume of 1 to 2 μL; reactions were performed in triplicates. Glyceraldehyde 3-phosphate dehydrogenase (GAPDH), Cyclophilin A, and beta-Actin served as standard housekeeping genes; 16S-RNA and bacterial GAPDH were used as housekeeping genes for determination of fecal bacterial gene expression. PCR conditions for all reactions were: 95 °C for 3 minutes, followed by 40 cycles of 95 °C for 30 seconds, 55 °C for 30 seconds, and 72 °C for 30 seconds. Primers and primer sequences were retrieved from the existing internal primer stock, from papers, collaborators, or designed via Primer-Blast (NCBI). All primer pairs were re-evaluated for self-complementation and gene specificity via blasting on Primer-Blast (NCBI). Primer amplification efficiencies were tested by generating a standard curve from PCR reactions of serial dilutions of the cDNA of positive control tissues. Furthermore, the melting curve of the primers was checked for quality control. Primer sequences of genes of interest and of housekeeping genes for fecal bacterial gene expression are listed in Supplementary Table 1.

### Microarray Analysis

Total RNA from GEJ and forestomach tissues from 12-month-old L2-IL1B WT and L2-IL1B-FXR KO mice (n = 3) were extracted by TRIzol reagent (Invitrogen) according to the manufacturers protocol. Expression profiling was accomplished using Mouse gene 2.1 Affymetrix GeneChip expression arrays. Differential expression was determined using Limma^51^ as implemented in oneChannelGUI,^52^ operating as part of the Bioconductor Suite^53^ in the R statistical computing environment.^54^ Estimates of the statistical significance of overlap between gene sets were performed using the χ^2^ as implemented in R. Raw data have been deposited in the National Center for Biotechnology Information’s Gene Expression Omnibus (GEO) (GSE103616). Functional annotation of phenotypes was performed by gene set enrichment analysis (GSEA) using the MSigDB database v5.2, where each phenotype was represented by 3 mice.

### High-throughput 16S rRNA Gene Amplicon Sequencing and Analysis

16S rRNA sequencing from patients’ stool, saliva, and PAXgene tissue samples was performed to characterize the gut, oral, and local esophageal microbial microenvironment of the human patient cohort in different disease states. 16S rRNA gene sequencing from experimental mice on different diets and treatments was performed from cecal content and fecal samples to characterize the diet- and treatment-dependent microbial shifts in the mice. Therefore, DNA was extracted from the samples using different extraction methods.

### DNA Extraction From Fecal Samples for 16S rRNA Gene Amplicon Sequencing

DNA from human fecal samples was extracted using a short modified version of the previously published Godon protocol.^55^ Feces were already frozen with DNA stabilizer (Sarstedt, Stool Collection Tubes with Stool DNA Stabilizer, Cat. 1038111300). To 700 μL of the feces-DNA stabilizer mix, 250 μL 4M guanidinium thiocyanate and 500 μL 5% N-laurolylsarcosine were added. The probes were incubated for 1 hour at 70 °C. Sterile silica beads (0.1 mm, Biospec products) were used for bacterial cell lysis in a FastPrep-24 bead beater (MP Biomedicals). Then, 15 mg polyvinylpyrrolidone was added, and the suspension was centrifuged at 15,000 g at 4 °C for 3 minutes. The supernatant was collected and again centrifuged at 15,000 g at 4 °C for 3 minutes. Thereafter, 500 μL of clear supernatant was collected, and 5 μL RNAse (10 mg/mL) were added to the samples, followed by an incubation step for 20 minutes at 37 °C and shaking at 700 rpm. Subsequently, DNA was extracted with the NucleoSpin gDNA Clean-up kit following the manufacturer’s protocol. DNA from human PAXgene biopsy samples was extracted using the PAXgene tissue DNA-Kit (Preanalytix), following the manufacturer’s protocol for purification of genomic DNA from sections of PAXgene-treated, paraffin-embedded tissue. For all extracted DNA samples, a sodium acetate precipitation was performed for purification and concentration of the samples. DNA samples were mixed 1:10 with a sodium acetate solution (3M). Then, 4 volumes of 100% ethanol were added, mixed thoroughly, and incubated over night at −20 °C. All samples were centrifuged at top speed for 30 minutes at 4 °C, and the supernatant was discarded. The pelleted DNA was washed with 500-μL ice-cold 80% ethanol. The samples were centrifuged at top speed for 10 minutes at 4 °C, and the supernatant was discarded. If needed, the washing step was repeated once. The DNA pellet was air-dried and resuspended in 10 μL nuclease-free dH2O. After precipitation, concentration and purity of all samples was measured on a Nanodrop 1000 Spectrophotometer (Thermo Scientific). In a test-sequencing experiment, it was shown that DNA probes had sufficient quantitiy and quality for further analyses.

The V3/V4 region of 16S rRNA genes was amplified (25 cycles for fecal samples, 15 cycles for tissue biopsies) from 12 ng of metagenomic DNA using the bacteria-specific primers 341F and 785R following a 2-step procedure to limit amplification bias.^56^ Amplicons were purified using the AMPure XP system (Beckmann), pooled in an equimolar amount, and sequenced in paired-end modus (PE275) using a MiSeq system (Illumina, Inc) following the manufacturer’s instructions.

### Analysis of 16S rRNA Sequencing Data

16S rRNA sequencing data were analyzed using IMNGS, a web-based pipeline for processing of 16SRNA amplicon datasets,^57^ and RHEA, an R-based pipeline for data analysis and visualization. Beginning with the IMNGS workflow, resulting sequences were remultiplexed with a Perl script provided by the inventors of IMNGS termed remultiplexor.^57^ The IMNGS workflow itself is based on the UPARSE pipeline.^58^ In the IMNGS workflow, pairing, quality filtering, and clustering of zero radius operational transcriptional units (zOTUs) were performed, wherefore USEARCH 8.0 was used.^59^ Therefore, all reads were trimmed to the position of the first base with a quality score smaller than 3 and then paired. The resulting sequences were size filtered and sequences with assembled size <300 and >600 nucleotides were excluded. Paired reads with an expected error bigger than three were also excluded. Remaining sequences were trimmed by 5 nucleotides on each side to avoid guanine-cytosine (GC) bias and nonrandom base composition. After processing of the remultiplexed data by the IMNGS workflow, a zOTU-table with associated sequences and taxonomic information for further analysis was generated. Additional analyses, including normalization, alpha- and beta-diversity, taxonomic abundance, and correlation were performed using the Rhea-pipeline created for the R-interface RStudio.^60^, ^61^, ^62^

### Operational Transcriptional Unit Clustering and Correlation Analysis of Human Stool Samples

Raw sequencing reads of the 16S-V4 region were analyzed using a previously described pipeline. Briefly, an operational transcriptional unit (out) count per sample table was generated using USEARCH v11.0.667,^59^ and taxonomies were assigned using RDP classifier trained with 16S rRNA training set 18.^63^ The samples with less than 3000 reads were filtered out. For each bacteria family, a Pearson correlation was calculated between its relative abundance and CA, DCA, and TUDCA levels across all samples.

### Mass spectrometry for Targeted BA Analysis

Serum samples from the L2-IL1B and L2-IL1B-FXR KO mouse cohort, CD, HFD, and HFD + OCA-treated L2-IL1B mice and healthy control individuals and patients diagnosed with BE, dysplasia, and EAC from the BarrettNET study were used for metabolomic analyses. Also, cecal content and feces from CD, HFD, and HFD + OCA-treated L2-IL1B mice and stool of patients were submitted for metabolomic analysis. Around 20 mg cecal and fecal/stool content or tissue were weighed in 2-mL bead beater tubes (CKMix 2 mL, Bertin Technologies) filled with ceramic beads (1.4 mm and 2.8 mm ceramic beads i.d.). Samples were later normalized to input weight.

For mouse samples, 1 mL methanol-based dehydrocholic acid extraction solvent (c = 1.3 μmol/L) as an internal standard for work-up losses was added. For human samples, 100 mg of stool was extracted with 5 mL methanol-based dehydrocholic acid. Samples were homogenized using a bead beater (Precellys Evolution, Bertin Technologies) supplied with a Cryolys cooling module (Bertin Technologies, cooled with liquid nitrogen;3 × 20 seconds at 10.000 rpm, 15-second breaks). The suspension was centrifuged (10 minutes, 8000 rpm, 10 °C) using an Eppendorf Centrifuge 5415R (Eppendorf). For metabolomic analysis of serum samples, 30 μL of serum was diluted with 270 μL methanolic dehydrocholic acid solution. After centrifugation as described above, the supernatant was used for analysis.

Targeted metabolomic analysis of BAs was performed according to a method published by Reiter et al.^64^ Briefly, 20 μL of isotopically labeled BAs (ca. 7 μM each) were added to 100 μL of sample extract. Targeted BA measurement was done using a QTRAP 5500 triple quadrupole mass spectrometer (MS) (Sciex) coupled to an ExionLC AD (Sciex) ultrahigh performance liquid chromatography (LC) system. A multiple reaction monitoring (MRM) method was used for the detection and quantification of the BAs. An electrospray ion voltage of −4500 V and the following ion source parameters were applied: curtain gas (35 psi), temperature (450 °C), gas 1 (55 psi), gas 2 (65 psi), and entrance potential (−10 V). The MS parameters and LC conditions were optimized using commercially available standards of endogenous BAs and deuterated BAs, for the simultaneous quantification of selected 34 analytes. For separation of the analytes, a 100 × 2.1 mm, 100 Å, 1.7 μm, Kinetex C18 column (Phenomenex) was used. Chromatographic separation was performed with a constant flow rate of 0.4 mL/min using a mobile phase consisted of water (eluent A) and acetonitrile/water (95/5, v/v, eluent B), both containing 5 mM ammonium acetate and 0.1% formic acid. The gradient elution started with 25% B for 2 minutes, increased at 3.5 minutes to 27% B, in 2 minutes to 35% B, which was held until 10 minutes, increased in 1 minute to 43% B, held for 1 minute, increased in 2 minutes to 58% B, held 3 minutes isocratically at 58% B. Then the concentration was increased to 65% at 17.5 minutes, with another increase to 80% B at 18 minutes, following an increase at 19 minutes to 100% B which was held for 1 minute, At 20.5 minutes, the column was equilibrated for 4.5 minutes at starting. The injection volume for all samples was 1 μL, the column oven temperature was set to 40 °C, and the auto-sampler was kept at 15 °C. Data acquisition and instrumental control were performed with Analyst 1.7 software (Sciex). Targeted metabolomic analyses were analyzed as BA intensities, or respectively, if correlated to input weight and dilution, in nmol/g feces. BAs detected via targeted BA analysis are listed in Supplementary Table 2.

### Analysis and Statistical Evaluation of Targeted Metabolomic Analyses

For single BA analyses, values of the row data were first multiplied by 1000 and then log2 transformed. BA with N/A values were not considered. The data of all other BAs were transferred to GraphPad for statistical analysis. Targeted serum BA analysis comparing CD with HFD mice were visualized as violin plots showing BA intensities between samples, as well as a correlation heatmap of BA levels with microbial bacteria in the gut. For further analysis and visualization, including partial least squares – discriminant analysis (PLS-DA), and clustered heatmap, the web-based tool metaboanalyst was employed.^65^

### Organoid Culture, Maintenance, and Experiments

#### Preparation of conditioned medium for organoid maintenance

L-WRN (LWnt3A, R-spondin 3, and Noggin) conditioned medium with additional growth factors was used as growth medium for organoid culture. Media were conditioned wit L-WRN-producing cells (ATCC CRL3276) according to the manufacturer's protocol. The complete growth medium consisted of advanced Dulbecco's Modified Eagle's Medium (DMEM)/F12, supplemented with 10% fetal bovine serum (FBS), 1% Penicillin/Streptomycin, HEPES buffer (pH 7.8), and Glutamax (all Gibco via ThermoFisher). For selection, G-418 and Hygromycin B (all Gibco via ThermoFisher) were used. For organoid culture, L-WRN conditioned media was supplemented with additional growth factors, including 1xB27 and N2 (17504044, 17502048, ThermoFisher), 50 ng/mL human EGF (AF-100-15, Peprotech), and 1mM N-acetyl cysteine (A7250, Sigma Aldrich).

#### Isolation and maintenance of murine organoids

Mouse organoid culture was performed according to the procedure published by Pastula et al with minor adjustments.^66^ Resected and cleaned cardia tissue was cut into small pieces using surgical scissors and transferred to an Eppendorf tube containing 200-300 μl Accutase cell detachment solution (A6964, Sigma-Aldrich). The tissue was incubated in Accutase on a shaker for 15 minutes for enzymatic tissue digestion. Tissue pieces were transferred to a 50-mL collection tube containing 20 mL of ice-cold Dulbecco’s phosphate buffered saline (dPBS) (14190144, Gibco via ThermoFisher) supplemented with 2 mM EDTA (AM9260G, Invitrogen via ThermoFisher) and EGTA (3054.2, Roth) each. All following steps were performed on ice. The tissue was incubated on a shaker on ice for 45 minutes for chemical tissue digestion. Supernatant was removed from sedimented tissue pieces, and tissue was washed and mechanically disintegrated by pipetting up and down in 10 mL of cold dPBS (14190144, Gibco via ThermoFisher) + 10% FBS (10500064, Gibco via ThermoFisher). The tissue suspension was passed through a 70-μm cell strainer into a 50-mL tube. The tissue was removed from the strainer by washing with 10 mL of fresh dPBS + 10% FBS. Mechanical disintegration, filtration, and recovery of the tissue from the strainer as described was repeated 4 times. The final volume of cell suspension was centrifuged at 4 °C for 10 minutes at 400 g. The supernatant was removed, and the cells were resuspended in 150 to 300 μL Matrigel (354230, Corning). Then, 50 μL ice-cold Matrigel per well were plated in a 24-well plate prewarmed to 37 °C and overlaid with 500 μL of complete growth media per well after solidification. The plate was incubated at 37 °C. Media was changed every 2 to 3 days, and organoids were passaged every 7 to 10 days depending on the growth rate. Media was removed, and Matrigel was disrupted by repetitive pipetting with ice-cold dPBS + 10% FBS buffer, and the suspension from each well was pooled into a 15-mL collection tube. The washing step was repeated. Matrigel and organoids were gently disrupted by repetitive pipetting of the suspension in the 15-mL collection tube. The tube was then centrifuged at 4 °C for 10 minutes at 400 g. Afterwards, the supernatant and Matrigel debris were carefully removed. The cell pellet was resuspended in fresh Matrigel, plated into a prewarmed 24-well plate, and overlaid with media as previously described. Wells were expanded 1:2 to 1:4 per passage depending on the growth rate of the organoids.

#### Isolation and maintenance of human organoids

Organoids were isolated from biopsies taken from patients with BE and GEAC. Prior to biopsy collection, explicit informed consent was obtained from all patients in accordance with institutional guidelines and ethical standards (FREEZE-BiObank).

Freshly taken biopsies in tissue storage solution (Miltenyi Biotec) + 10 μM Y-27632/Rock Inhibitor were transferred and digested in a FALCON containing prewarmed 10 mL digestion medium (DMEM+++ composed of advanced DMEM/F12, 1% penicillin/streptomycin, 1% HEPES buffer, and 1% Glutamax, together with 1% Primocin, 1 mg of Collagenase XI 1, and 10 mg of Dispase II). The biopsy was incubated for 30 to 90 minutes in a water bath (37 °C) and vigorously shaken to promote tissue disruption and release of glands and crypts during digestion. After incubation step, FALCON was centrifuged (5 minutes, 400 g, room temperature), supernatant was discarded, 15 mL digestion buffer was added, and sample was centrifuged again. This washing step was repeated 3 times. After the last washing step, tissue pellets were resuspended in 100% MG and plated as 20-μL domes. After the solidification of domes for 10 minutes, the medium (DMEM+++ containing 1xB27, 1xN2, 50 μg/mL human EGF, 1 mM N-Acetylcystein, 10 mM Nicotinamide, 5 μM P38 MAPK Inhibitor, 2 μM TGF beta inhibitor, 0.5 μM CHIR99021, 10 nM Gastrin, 100 ng/mL FGF-10, 10 μM Rock-Inhibitor, 100 μg/mL Primocin) was added.

#### Treatment of organoids with BAs

Mouse cardia organoids were isolated from L2-IL1B mice. Treatment was started 2 days after the third to fourth passage of the organoids. The organoid treatment was performed for 72 hours with OCA, DCA or TβMCA, or OCA + DCA, or OCA + TβMCA in concentrations of 10 and 100 μM diluted in the medium, respectively. Every 24 hours, cell numbers were counted and cultures photographed, and medium was changed subsequently. After 72 hours, 2 to 3 wells with organoids were pooled, and RNA was extracted using the RNeasy Micro Kit (Qiagen), and conditioned media from every treatment condition was collected and stored. The number of organoids counted every 24 hours was evaluated as percentage of organoid numbers relative to 0 hours.

#### Histologic analysis of organoids

For fixation and embedding of organoids for FFPE sections, organoids were grown on cover slips placed into a 24-well plate. Organoids were washed 3 times with 500 μL of PBS^+^ (dPBS + 0.9mM CaCl_2_ and 0.493 mM of MgCl_2_) per well for one minute before fixation in 500 μl of 4% paraformaldehyde (PFA) for 30 minutes at room temperature on a shaker. After fixation, washing steps were repeated. Organoids were transferred to Bio-Net histology cassettes (09-0403, Langenbrinck GmbH), dehydrated, paraffin-embedded, and cut into sections using a manual microtome. Staining and IHC were performed as for tissue sections. Quantification was assessed as the number of positive cells compared with all cells per organoid. All organoids from one mouse, which had been subjected with the same treatment, were counted as technical replicates. Organoids from different mice were defined as biological replicates. Organoids from n = 3 mice per condition were used for statistical analysis. To this end, ordinary 1-way ANOVA with Tukey test to correct for multiple comparisons was performed.

### Immunofluorescence Staining of Paraffin-Embedded Organoid Slides

Human organoid samples (3 GEAC, 3 BE) were fixed after 2 to 4 passages using 4% PFA in phosphate-buffered saline (PBS) with a pH of 7.4 (sc-281692, ChemCruz via Santa Cruz Biotechnology) for 30 minutes at room temperature. Fixed organoids were dehydrated using graded ethanol series and xylene, and were finally embedded in paraffin blocks using a standard tissue processing protocol.

FFPE organoid blocks were cut into 3.0-μm-thick sections using a microtome. Sections were mounted onto positively charged glass slides and dried at room temperature overnight. Slides were immersed in xylene for 15 minutes (2 times) to remove paraffin. Sections were rehydrated through a series of graded ethanol solutions (100%, 95%, and 70%). Slides were subjected to heat-mediated antigen retrieval using a target retrieval solution, pH 9.0 (S2367, Dako) in a pressure cooker for 15 minutes. After cooling, slides were washed with 1 × wash buffer (S3006, Dako).Then slides were blocked with 10% goat serum (ENG9010-10, BIOZOL Diagnostica Vertrieb GmbH), 1:100 Fc block anti CD16/CD32 (553142, BD Biosciences) in antibody diluent with background reducing components (S3022, Dako) for 1 hour at room temperature to minimize nonspecific binding. Next, sections were incubated with the primary antibody to FXR (sc-13063, Santa Cruz Biotechnology) in antibody diluent (1:25) with background reducing compounds overnight at 4 °C in a humidified chamber. Slides were washed 3 times for 5 minutes each with 1× wash buffer to remove unbound primary antibodies. Fluorescent-labeled secondary antibodies 1:400 (A11034, Invitrogen) were applied to the sections for 1 hour at room temperature, then slides were washed 3 times more. To minimize autofluorescence of samples, we used Quenching Kit (SP-8500, Vector Laboratories Inc). As a final step of this kit, slides were mounted using an anti-fade medium with 4′,6-diamidino-2-phenylindole (DAPI) to preserve fluorescence signals and counterstain nuclei.

Per 1 sample, 5 different fields of view (20× magnification) containing organoids were taken. Images were analyzed with CellProfiler 4.2.5∗. The nuclear signal of FXR was measured in the regions of interests defined by the DAPI signal. Nuclei were classified as positive if their mean FXR intensity exceeded that of 75% of all nuclei. The result was expressed as the percentage of positive nuclei relative to the total number of nuclei in field of view.

### RNA Extraction and Downstream Applications

In brief, 3 to 6 wells of organoids were harvested with 500 μL of dPBS + 10% FBS per well; cells were pooled and centrifuged for 10 minutes at 4 °C and 400 rcf. The supernatant was removed, and the pellet was resuspended in 500 μL collagenase/ dispase (1 mg/mL) and incubated at 37 °C for 1 hour to digest Matrigel residuals enzymatically. The reaction was stopped with 500 μL 5 mM EDTA (AM9260G, Invitrogen via ThermoFisher) in dPBS. The tube was filled 10 mL with dPBS, centrifuged for 10 minutes at 4 °C and 400 rcf, and the supernatant was removed. The remaining cell pellet was lysed in 600 μL RLT-buffer (Lysis buffer; 1015762; Qiagen) supplemented with 1 % beta-mercaptoethanol (4227.3, Roth). RNA isolation was performed using the RNeasy Mini Kit (74104; Qiagen) according to the manufacturer’s protocol. Reverse transcription PCR and qRT-PCR were both conducted as described for tissue samples.

### DNA Damage Enzyme-linked Immunosorbent Assay

OxiSelect Oxidative DNA ***Damage*** enzyme-linked immunosorbent assay (ELISA), 8′OhdG Quantitation (Cell Biolabs, Inc. STA-320) was performed according to the manufacturer’s protocol using conditioned media from the different treatments done in the organoid cultures. Conditioned media from 12-month-old L2-IL1b mouse organoids treated with OCA, DCA, TβMCA, OCA+DCA, or OCA+TβMCA and without treatment were analyzed after 72 hours of treatment, respectively. Data was acquired on a multiskan FC microplate reader (Thermo Scientific) and analyzed using Microsoft Excel and GraphPad Prism version 8.
