# Supplementary material for: Loss of FXR or Bile Acid-dependent Inhibition Accelerate Carcinogenesis of Gastroesophageal Adenocarcinoma
Source: Cell Mol Gastroenterol Hepatol. 2025 Mar 24;19(8):101505. doi: 10.1016/j.jcmgh.2025.101505 (PMC12152668; doi:10.1016/j.jcmgh.2025.101505)
Supplement: Supplementary Tables 1 and 2 [file mmc1.pdf]

Supplementary Table 1. Primers for Quantitative Real Time - PCR

Target		Sequence	Melting T.	Notes
FXR	Forward	5´-GGCTGCAAAGGTTTCTTCCG-3´	67.9	
	Reverse	5´-ACATTCAGCCAACATCCCCA-3´	67.6	
IBABP	Forward	5´-CACCATTGGCAAAGAATGTG-3´	65.7	
	Reverse	5´-AACTTGTCACCCACGACCTC-3´	66.7	
SHP	Forward	5´-AGCTGGGTCCCAAGGAGTAT-3´	63.7	
	Reverse	5´- CTTGAGGGTAGAGGCCATGA -3´	64.1	
GAPDH	Forward	5´-GACATCAAGAAGGTGGTGAAGCAG-3´	68	
	Reverse	5´-ATACCAGGAAATGAGCTTGACAAA-3´	64.5	
βactin	Forward	5´-CCGTGAACCCTAAGGCCAACC-3´	72.7	
	Reverse	5´-ACCCCGTCTCCGGAGTCCATC-3´	69.5	
BSH	Forward	5´-ATGGGCGGACTAGGATTACC-3´	63.8	Working conc. 100 µM
	Reverse	5´-TGCCACTCTCTGTCTTC-3´	54.2	
16S-RNA	Forward	5´-TGATCCTGGCTCAGGACGAA-3´	68.3	Working conc. 100 µM
	Reverse	5´-TGCAAGCACCAATCAATACCA-3´	66.2	

Supplementary Table 2. Bile Acids measured in targeted BA metabolomic analysis

5β-Cholic acid-3α-ol-7-one, 7-Ketolithocholic acid, (7-KLCA)	3-Dehydrocholic acid (3-DHCA)
5β-Cholic acid-3α-ol-12-one, 12- Ketolithocholic acid, (12-KLCA)	5β-Cholic acid-3α-ol-6,7-dione, (6,7- DKLCA)
α-Muricholic acid, (α-MCA)	5β-Cholic acid-3α-ol-6-one, (6-KLCA)
β-Muricholic acid, (β-MCA)	7-Dehydrocholic acid, (7-DHCA)
5β-Cholen-24-oic acid-3,12-diol, Apocholic acid, (ApCA)	Cholic acid-7-sulphate, (7-SCA)
Chenodeoxycholic acid, (CDCA)	12-Dehydrocholic acid, (12-DHCA)
Cholic acid, (CA)	5β-Cholic acid-7α-ol-3-one, (Ca-7ol3one)
Deoxycholic acid, (DCA)	Glycocholic acid, (GCA)

Glycochenodeoxycholic acid, (GCDCA)	Glycoursodeoxycholic acid, (GUDCA)
Glycodeoxycholic acid, (GDCA)	Isolithocholic acid, (ILCA)
Hyodeoxycholic acid, (HDCA)	Taurocholic acid, (TCA)
Lithocholic acid, (LCA)	Taurohyodeoxycholic acid, (THDCA)
Tauro-α-Muricholic acid, (T-α-MCA)	Taurolithocholic acid, (TLCA)
Taurochenodeoxycholic acid, (TCDCA)	Tauro-ω-Muricholic acid, (T-ω-MCA)
Taurodeoxycholic acid, (TDCA)	Allocholic acid, (ACA)
Tauroursodeoxycholic acid, (TUDCA)	Ursocholic acid, (UCA)
Ursodeoxycholic acid, (UDCA)	5β-Cholic acid-3α-ol-7,12-dione, (7,12-DKLCA)
Glycolithocholic acid, (GLCA)	Dehydrolithocholic acid, (DHLCA)
Murideoxycholic acid, (MDCA)	Glycohyocholic acid, (GHCA)
Allolithocholic acid, (ALCA)	Lithocholenic acid, (LCenA)
Glycohyodeoxycholic acid, (GHCA)	Obeticholic acid, (OCA)
Isodeoxycholic acid, (IDCA)	γ-Muricholic acid / Hyocholic acid, (γMCA)
